# Synthesis, DFT Calculations, and In Vitro Antioxidant Study on Novel Carba-Analogs of Vitamin E

**DOI:** 10.3390/antiox8120589

**Published:** 2019-11-26

**Authors:** Aneta Baj, Jakub Cedrowski, Ewa Olchowik-Grabarek, Artur Ratkiewicz, Stanislaw Witkowski

**Affiliations:** 1 Faculty of Chemistry, University of Bialystok, Ciolkowskiego 1K, 15-245 Bialystok, Poland; aneta.baj@uwb.edu.pl (A.B.); artrat@uwb.edu.pl (A.R.); 2 Faculty of Chemistry, University of Warsaw, Pasteura 1, 02-093 Warsaw, Poland; jcedrowski@chem.uw.edu.pl; 3 Faculty of Biology, University of Bialystok, Ciolkowskiego 1J, 15-245 Bialystok, Poland; ewaolch@uwb.edu.pl

**Keywords:** vitamin E, Trolox, carba-analogs, antioxidant activity

## Abstract

Vitamin E is the most active natural lipophilic antioxidant with a broad spectrum of biological activity. α-Tocopherol (α-T), the main representative of the vitamin E family, is a strong inhibitor of lipid peroxidation as a chain-breaking antioxidant. Antioxidant and antiradical properties of vitamin E result from the presence of a phenolic hydroxyl group at the C-6 position. Due to stereoelectronic effects in the dihydropyranyl ring, the dissociation enthalpy for phenolic O–H bond (BDE_OH_) is reduced. The high chain-breaking reactivity of α-T is mainly attributed to orbital overlapping of the 2p-type lone pair on the oxygen atom (O1) in para position to the phenolic group, and the aromatic π-electron system. The influence of the O1 atom on the antioxidant activity of vitamin E was estimated quantitatively. The all-*rac*-1-carba-α-tocopherol was synthesized for the first time. Along with model compounds, 1-carba-analog of Trolox and its methyl ester were screened for their in vitro antioxidant activity by inhibition of styrene oxidation, and for the radical-reducing properties by means of 2,2-diphenyl-1-picrylhydrazyl free radical (DPPH) scavenging assay. To study the antioxidant activity, density functional theory (DFT) was also applied. Reaction enthalpies related to HAT (hydrogen atom transfer), SET–PT (sequential electron transfer—proton transfer), and SPLET (sequential proton loss—electron transfer) mechanisms were calculated.

## 1. Introduction

**Figure 1:**
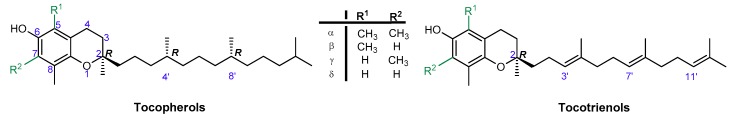
Chemical structures of tocopherols (T) and tocotrienols (T3).

Vitamin E is one of the most effective natural lipophilic chain-breaking antioxidants, which exhibits also a broad spectrum of other biological functions. Due to lipophilic properties vitamin E incorporates into biological membranes and protects them from oxidative damage. Moreover, it regulates the structural and functional properties of cell membranes, giving them adequate fluidity, stability, and permeability. The term “vitamin E” is a collective name of eight naturally occurring compounds with similar structures, called tocochromanols. The vitamin E family includes four tocopherols with a saturated phytyl side-chain and four tocotrienols with a side-chain with three double bonds at the 3’, 7’, and 11’ positions (farnesyl chain). Both groups consist of four forms: α-, β-, γ-, and δ-, differing in location and number of methyl groups on the aromatic ring (Figure 1). α-Tocopherol (α-T) shows the highest antioxidant activity [1,2,3]. 

**Figure 2:**
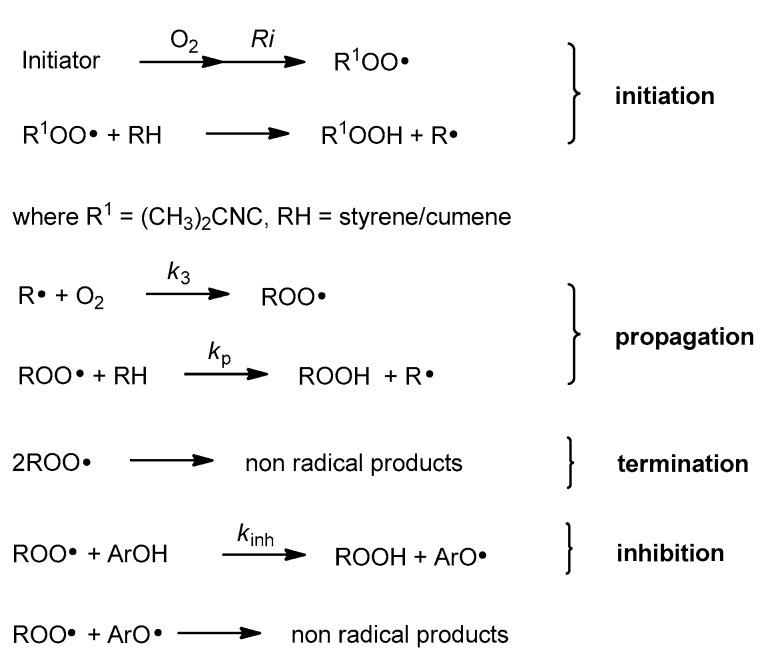
Chain reaction mechanism of initiated autoxidation. Propagation steps are repeated from several to hundreds of times. *R*_i_, rate of chain initiation [Ms^−1^]; *k*_3_, rate constant of formation of alkyl peroxyl radical [M^−1^ s^−1^]; *k*_p_, propagation rate constant [M^−1^ s^−1^].

Antioxidant activity of vitamin E was reported for the first time in 1937 [4]. In vitro studies, conducted in the 1980s by Ingold and Burton, showed that α-T used at low concentration is one of the best chain-breaking antioxidants from all phenolic antioxidants known [5]. Phenolic antioxidants (ArOH) are able to stop/break the propagation process by direct reaction with peroxyl radicals (ROO●) (Figure 2). In general, mono-phenolic chain-breaking antioxidants can trap up to two peroxyl radicals [6,7]. α-T has two characteristics of an effective chain-breaking antioxidant. Firstly, it can rapidly transfer the phenolic H atom onto a lipid peroxyl radical (LOO●). Secondly, the tocopheroxyl radical (T●) is unreactive towards O_2_ or polyunsaturated lipids. Furthermore, T● can be further oxidized by another LOO● to quinone form or reduced back to phenol by co-antioxidants (e.g., ascorbic acid, *β*-carotene, or coenzyme Q) [8]. If the value of the inhibition rate constant *k*_inh_ [M^−1^ s^−1^] is at least three orders of magnitude higher than the propagation rate constant *k*_p_ [M^−1^ s^−1^], such antioxidants can be considered as effective “chain-breaking” antioxidants in the system tested [9]. The values of *k*_p_ for autoxidation of styrene and cumene in chlorobenzene at 30 °C are 41 M^−1^s^−1^ and 0.32 M^−1^ s^−1^, respectively [10,11]. The *k*_inh_ for α-T ranges from 6 × 10^3^ M^−1^ s^−1^ in phospholipid bilayers to 3 × 10^6^ M^−1^ s^−1^ in organic solvents. One molecule of α-T can destroy two radicals and terminate two potential radical chains [12,13].

Prooxidant activity of vitamin E has also been reported [14,15]. This kind of α-tocopherol activity was observed during the oxidation of low-density lipoprotein (LDL) [16]. When the antioxidant network is balanced, the prooxidant activity of vitamin E radicals is inhibited by co-antioxidants, which can reduce the radicals formed back to vitamin E. Due to this dichotomy of the antioxidant and prooxidant nature of vitamin E, it is often called “Janus molecule” [17].

**Figure 3:**
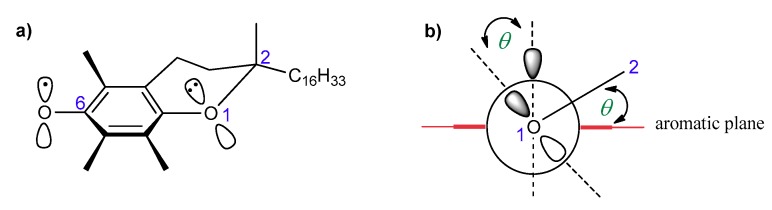
(**a**) Structure of T● with the numbering of atoms; (**b**) stabilization effect of T●. *θ*, the angle between the 2p-orbital on the O1 atom and the perpendicular to the plane of the aromatic ring.

According to Ingold and Burton, the high antioxidant activity of vitamin E should be attributed to the high reactivity of the phenolic group at the C-6 position, which is caused by resonance stabilization from the stereoelectronic effects of the respective chromanyloxyl radicals formed [5,18,19]. The dihydropyranyl ring has a half-chair conformation and forces almost perpendicular orientation of the 2p-type orbital of the oxygen atom in position 1 to the aromatic ring plane (angle *θ* approx. 16°, Figure 3). Electron delocalization induces a strong overlap between the 2p-type orbital on the O1 atom and the aromatic π-electron system. The smaller the angle *θ* between the p-orbital and the axis perpendicular to the plane of the aromatic ring, the greater the overlap of the orbitals, and thus, the lower the binding strength of the O–H phenolic group, and therefore the higher the activity of a phenolic antioxidant [5,12,13,19,20]. Ingold and Burton estimated the antioxidant activity of tocopherols and selected phenols based on the rate of inhibition of styrene autoxidation (*k*_inh_). The results are listed in Table 1. They concluded that high chain-breaking activity arises mainly from the aforementioned stereoelectronic effects exerted by 2p-type lone pair of electrons of the heterocyclic oxygen atom O1 situated in the para position to the phenolic group, which is able to stabilize the incipient phenoxyl radical. Additional factors include the electron-donating methyl substituents on the aromatic ring. β-, γ-, and δ-Tocopherols show a lower *k*_inh_ than fully methylated α-T. Nevertheless, the electron-donating or electron-withdrawing substituents found at C-2 and adjacent to the O1 atom increase or decrease antiradical properties, respectively. Thus, carboxyl group reduces the contribution of non-binding electron pairs of the heterocyclic oxygen atom in the stabilization of phenoxyl radicals. Trolox (**1a**) shows less antioxidant activity than Trolox methyl ester (**1b**), 2,2,5,7,8-pentamethylchroman-6-ol (PMHC; **1c**), and α-tocopherol (Table 1) [5]. Reduction of the heterocyclic ring size to five-membered in benzofuran analog **2d** caused flattening of the heterocyclic ring and a more perpendicular orientation (*θ* approx. 6°) of the oxygen 2p-type orbital, which resulted in a significant increase in antioxidant activity compared to α-T (about 1.5 times) [18].

Comparison of the activity of α-T and 4-methoxy-2,3,5,6-tetramethylphenol (**3c**, deprived of the heterocyclic ring) showed a reduction of *k*_inh_ value from 32 × 10^5^ to 3.9 × 10^5^ M^−1^∙s^−1^. However, for phenol **3c**, the *k_inh_* value was similar to that for **3d** (pentamethylphenol). This can be explained by the fact that in **3c** the *p*-methoxy group is almost perpendicularly oriented to the aromatic plane (*θ* approx. 89%) due to steric hindrance of flanking ortho-methyl groups. In this orientation, the p-type orbital of the O1 atom is located with the aromatic plane and, as a consequence, the non-bonding electron pair cannot participate in the stabilization of the resulting phenoxyl radical. The above-mentioned observations prove that stereoelectronic factors are of great importance in the antioxidant and antiradical activity of the chroman-6-ol system [5].

**Figure 4:**
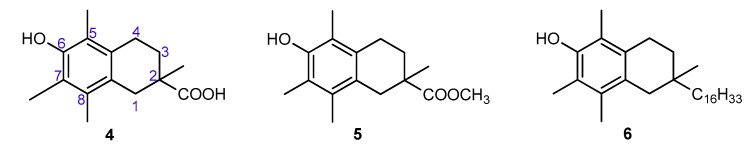
Chemical structures of the investigated 1-carba-analogs of vitamin E.

The aim of the present study was to precisely quantify the influence of the heterocyclic oxygen atom (O1) on the antioxidant activity of chroman-6-ols. Therefore, we decided to synthesize selected vitamin E analogs containing 1,2,3,4-tetrahydronaphthalene skeleton in place of the chromane ring, in which the oxygen atom would be substituted by “non-participating” carbon atom at position 1 (compounds **4**–**6**; Figure 4). Such synthetic manipulation should change the chemical and structural properties of the cyclic system (e.g., conformational preferences, electronic effects), which are important for antioxidant activity. Furthermore, the antioxidant tests on the model 1-carba-analogs provide more accurate data in the quantitative evaluation of stereoelectronic effects in the chroman-6-ols. These compounds are more structurally similar to the parent chromane system than the 4-methoxy-2,3,5,6-tetramethylphenol (**3c**), which was originally used as a reference by Ingold and Burton. The synthesis of carba-analogs **4** and **5** was reported earlier [21].

## 2. Materials and Methods

### 2.1. Chemical Synthesis

#### 2.1.1. General Information

All moisture and air-sensitive reactions were carried out under argon using oven-dried glassware. Starting materials and reagents were obtained from commercial sources and used without further purification. Solvents were dried by distillation over appropriate drying agents under argon atmosphere. Thin-layer chromatography (TLC) was performed on silica gel aluminum sheets (0.25 mm, silica gel 60 F254, Merck, Darmstadt, Germany), by spraying with ammonium molybdate/cerium(IV) sulfate solution, followed by heating. Flash chromatography (FC) was performed using 230–400 mesh silica gel (J. T. Baker, Center Valley, PA, USA). Melting points were determined by the capillary method using the MP70 Melting Point System (Mettler Toledo). ^1^H NMR (400 MHz) and ^13^C NMR (100 MHz) were recorded on a Bruker Avance II 400 MHz NMR spectrometer (Bruker GmbH, Mannheim, Germany). The chemical shifts (δ) are given in parts per million (ppm) relative to tetramethylsilane (TMS) using residual solvent protons as an internal standard. IR spectra were recorded using the Nicolet 6700 (KBr) or Nicolet Magna-IR 550 series II (ATR) FT-IR spectrometers (Thermo Fisher Scientific, Waltham, MA, USA). EI spectra were measured with the AMD-604 mass spectrometer (AMD Intectra GmbH, Harpstedt, Germany). ESI-HRMS spectra were obtained on the Agilent 6530 Accurate-Mass Q-TOF LC/MS system. 

All-*rac*-α-tocopherol (α-T) was obtained from all-*rac*-α-tocopheryl acetate via acid hydrolysis (methanol, conc. H_2_SO_4_), and was purified by FC (hexane/EtOAc, 12:1, *v*/*v*). All-*rac*-α-tocopheryl acetate (≥95%), farnesol (≥95%), and *R*,*S*-Trolox (**1a**, 97%) were purchased from Sigma-Aldrich Co. (Steinheim, Germany). The syntheses of compounds **1b** [22], **1c** [23], **4**, **5**, and **10** [21] were carried out according to the literature. Synthesis of phosphonium salt **9** is described in the Supplementary File S1.

#### 2.1.2. Synthesis of all-*rac*-1-carba-α-tocopherol (**6**)

##### (6-Methoxy-2,5,7,8-tetramethyl-1,2,3,4-tetrahydronaphthalen-2-yl)methanol (**11**)

LiAlH_4_ (0.124 g, 3.27 mmol) was added to a stirred solution of compound **10** (0.9 g, 3.26 mmol) in dry THF (30 mL) under argon atmosphere at 0 °C. After stirring for 18 h at room temperature (TLC control), the reaction mixture was poured into saturated NH_4_Cl solution (30 mL) and extracted with Et_2_O (3 × 30 mL). The organic layers were combined, washed with brine, dried over anhydrous Na_2_SO_4_, and evaporated in vacuo. The crude product was purified using FC (hexane/ethyl acetate, 7:2, *v*/*v*) to give compound **11** as a white powder (0.712 g, 88%). Mp 103.8–105.1 °C; *R*_f_ = 0.20 (hexane/ethyl acetate, 7:1, *v*/*v*); ^1^H NMR (400 MHz, CDCl_3_): δ 3.69 (s, 3H), 3.52–3.44 (m, 2H), 2.68–2.64 (m, 2H), 2.53 (d, *J* = 16.6 Hz, 1H), 2.39 (d, *J* = 16.6 Hz, 1H), 2.26, 2.20, 2.16 (3s, 9H), 1.79 (bs, 1H), 1.74–1.67 (m, 1H), 1.63–1.57 (m, 1H), 1.01 (s, 3H) ppm; ^13^C NMR (100 MHz, CDCl_3_): δ 154.29, 133.71, 132.84, 129.30, 126.51, 125.78, 71.37, 60.22, 36.47, 33.89, 30.17, 24.17, 22.31, 15.18, 12.72, 11.78 ppm; IR (KBr): 3315, 2915, 1456, 1247, 1108, 1042, 1012 cm^−1^; HRMS (ESI) *m/z*: [M+Na]^+^ calcd for C_16_H_24_O_2_Na 271.1674; found 271.1679.

##### 6-Methoxy-2,5,7,8-tetramethyl-1,2,3,4-tetrahydronaphthalene-2-carbaldehyde (**12**)

Dess-Martin periodinane (DMP, 1.336 g, 3.15 mmol) was added to a stirred solution of compound **11** (0.6 g, 2.42 mmol) in dry CH_2_Cl_2_ (15 mL) under argon at room temperature. After completion of the reaction (TLC control, 2 h), the mixture was quenched with water (20 mL) and extracted with CH_2_Cl_2_ (3 × 15 mL). The organic layers were combined, washed with saturated solution of NaHCO_3_ and brine, dried over Na_2_SO_4_, and evaporated under reduced pressure. The residue was purified by FC (hexane/ethyl acetate, 9:1, *v*/*v*) to give compound **12** (0.494 g, 83%) as a white solid. Mp 65.1–67.6 °C; *R*_f_ = 0.54 (hexane/ethyl acetate, 7:1, *v*/*v*); ^1^H NMR (400 MHz, CDCl_3_): δ 9.59 (s, 1H), 3.68 (s, 3H), 2.99 (d, *J* = 16.7 Hz, 1H), 2.69 (t, *J* = 6.7 Hz, 2H), 2.46 (d, *J* = 16.7 Hz, 1H), 2.26, 2.20, 2.17 (3s, 9H), 2.08–2.00 (m, 1H), 1.76–1.69 (m, 1H), 1.17 (s, 3H) ppm; ^13^C NMR (100 MHz, CDCl_3_): δ 205.74, 154.68, 133.60, 132.16, 127.78, 126.89, 125.88, 60.17, 44.87, 33.80, 28.64 23.90, 20.60, 15.20, 12.71, 11.74 ppm; IR (KBr): 2925, 2720, 1723, 1463, 1245, 1099, 1008 cm^−1^; HRMS (ESI) *m/z*: [M+Na]^+^ calcd for C_16_H_22_O_2_Na 269.1517; found 269.1526.

##### 6-Methoxy-2,5,7,8-tetramethyl-2-(4,8,12-trimethyltridec-1-enyl)-1,2,3,4-tetrahydronaphthalene (**13**)

LHMDS (solution in THF, 1.0 M, 1.5 mL, 1.5 mmol) was added dropwise to a suspension of compound **9** (0.714 g, 1.3 mmol) in dry THF (8 mL) under argon at −20 °C. After stirring for 30 min, a solution of compound **12** (0.25 g, 1.0 mmol) in dry THF (4 mL) was added and stirred for 30 min at −20 °C and at room temperature overnight. The reaction mixture was poured into saturated ammonium chloride solution and extracted with Et_2_O (3 × 15 mL). The crude product was purified by FC (hexane/ethyl acetate, 9:1, *v*/*v*) to give compound **13** as a colorless oil (0.399 g, 89%). *R*_f_ = 0.72 (hexane/CH_2_Cl_2_, 2:1, *v*/*v*); ^1^H NMR (400 MHz, CDCl_3_): δ 5.32–5.25 (m, 2H), 3.68 (s, 3H), 2.78 (d, *J* = 16.4 Hz, 1H), 2.64–2.62 (m, 2H), 2.78 (d, *J* = 16.4 Hz, 1H), 2.24, 2.17, 2.16 (3s, 9H), 2.03–1.98 (m, 2H), 1.65–1.09 (m, 17H), 1.24 (s, 3H), 0.91–0.87 (m, 12H) ppm; MS (EI) *m/z*: 440.2 (M^+^, 100%).

##### 6-Hydroxy-2,5,7,8-tetramethyl-2-(4,8,12-trimethyltridecyl)-1,2,3,4-tetrahydronaphthalene (**6**)

Hydrogenation. A mixture of compound **13** (0.269 g, 0.61 mmol) and 5% Pd/C (0.06 g) in ethyl acetate (15 mL) was stirred and hydrogenated under atmospheric pressure at room temperature. After total conversion (24 h), the reaction mixture was filtered through a pad of Celite and silica gel with hexane/CH_2_Cl_2_ (8:2, *v*/*v*) and the filtrate was evaporated under reduced pressure to give compound **14** as a colorless oil (0.243 g, 90%). *R*_f_ = 0.70 (hexane/CH_2_Cl_2_, 2:1, *v*/*v*); ^1^H NMR (400 MHz, CDCl_3_): δ 3.69 (s, 3H), 2.62 (t, *J* = 6.6 Hz, 2H), 2.46 (d, *J* = 16.5 Hz), 2.38 (d, *J* = 16.5 Hz, 1H), 2.26, 2.20, 2.16 (3s, 9H), 1.61–1.03 (m, 23H), 0.96 (s, 3H), 0.91–0.88 (m, 12H) ppm.

Demethylation. Boron trifluoride–dimethylsulfide complex (0.58 mL, 5.5 mmol, 10 eq) and anhydrous AlCl_3_ (0.366 g, 2.75 mmol) were added to a solution of compound **14** (0.243 g, 0.55 mmol) in dry CH_2_Cl_2_ (5 mL) and acetonitrile (3 mL) under argon at 0 °C. After stirring for 18 h at room temperature, the reaction mixture was concentrated under reduced pressure. Saturated NaHCO_3_ aqueous solution (20 mL) was added to the obtained residue, which was extracted with diethyl ether (3 × 30 mL). Combined organic phases were washed with brine and water and, dried over Na_2_SO_4_. The solvent was evaporated under reduced pressure and the crude product was purified by FC (hexane/ethyl acetate 10:1, *v*/*v*) to give compound **6** as a colorless oil (0.202 g, 86%). *R*_f_ = 0.46 (hexane/ethyl acetate, 9:1, *v*/*v*); ^1^H NMR (400 MHz, CDCl_3_): δ 4.56 (brs, 1H, OH), 2.65 (t, *J* = 6.5 Hz, 2H), 2.45 (d, *J* = 16.4 Hz, 1H), 2.37 (d, *J* = 16.4 Hz, 1H), 2.24 (s, 3H), 2.17 (s, 6H), 1.64–1.04 (m, 23H), 0.96 (s, 3H), 0.92–0.89 (m, 12H) ppm; ^13^C NMR (100 MHz, CDCl_3_): δ 149.29, 133.14, 132.51, 126.71, 119.40, 118.10, 41.57, 40.65, 40.62, 39.37, 38.08, 38.06, 37.99, 37.97, 37.50, 37.46, 37.41, 37.39, 37.28, 33.42, 33.40, 32.79, 32.77, 31.43, 27.97, 24.91, 24.80, 24.47, 24.46, 22.72, 22.63, 21.03, 19.75, 19.72, 19.69, 15.18, 12.31, 11.24 ppm; IR (ATR): 3479, 2922, 2866, 1458, 1375, 1209, 1099 cm^−1^; HRMS (ESI) m/z: [M-H]^-^ calcd for C_30_H_51_O 427.3940, found 427.3947.

### 2.2. Autoxidation Procedure

#### 2.2.1. Materials

Styrene (≥99%, Sigma-Aldrich Co, Steinheim, Germany) and cumene (98%, Sigma-Aldrich) were percolated on alumina and silica before the experiments in order to remove traces of stabilizer (4-*tert*-butylcatechol). Initiator, α,α’-Azobisisobutyronitrile (AIBN, >98% GC, Fluka Chemika, Buchs, Switzerland) was recrystallized from methanol before use. Solvents, chlorobenzene (99.9% HPLC grade, Sigma-Aldrich, St. Louis, MO, USA) and acetonitrile (≥99.9% HPLC grade, Sigma-Aldrich, Steinheim, Germany) were of the highest grade commercially available and used as received. 2,2,5,7,8-pentamethylchroman-6-ol (**1c**, PMHC, ≥99%, Sigma-Aldrich, St. Louis, MO, USA), an autoxidation inhibitor in the reference flask (see Section 2.2.3), was used as received.

#### 2.2.2. Measurements of Autoxidation Rate

Antioxidant activity toward peroxyl radicals (ROO●) was measured by studying autoxidation of styrene (4.3 M) and cumene (3.6 M) in chlorobenzene at 30 °C initiated by AIBN (0.05 M), in the presence of tested compounds (~3–8 μM). For each compound, the experiment was performed in triplicate. Autoxidation of styrene/cumene as model hydrocarbons is a chain mechanism reaction and could be described by the reactions presented in Figure 2.

#### 2.2.3. Methodology of Autoxidation Measurements

**Figure 5:**
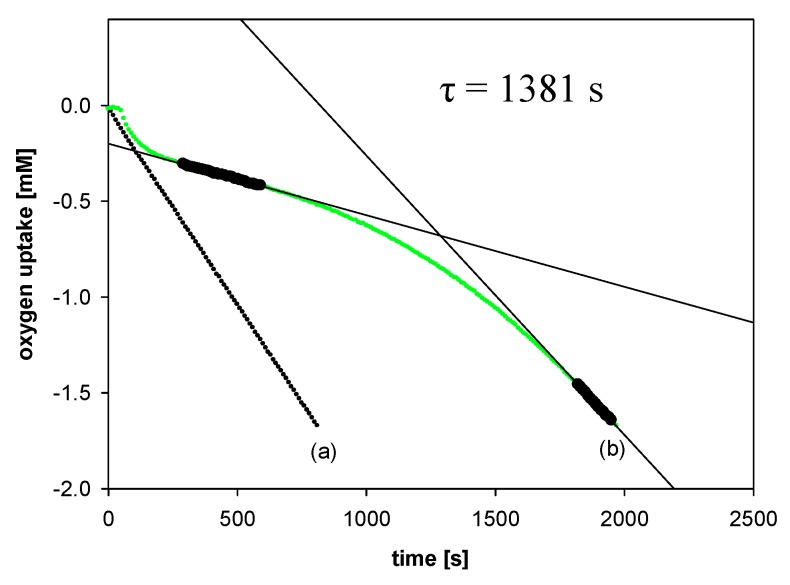
Oxygen uptake during the autoxidation of styrene (4.3 M) in THF, initiated by 0.05 M AIBN at 30 °C, in the presence of: (**a**) Without additions—process uninhibited; (**b**) PMHC (5.0 μM). t, time [s]; τ, the value of the induction period.

All experiments were performed by measuring the oxygen pressure changes in the two-channel gas uptake apparatus based on an ultra-precise differential pressure sensor (pressure transducer) described elsewhere [24,25]. Progress of oxidation was monitored by the measurement of the oxygen pressure difference between the reference (total solution volume ~4 mL) and sample flask (total solution volume ~4 mL)—in the sample flask, the oxygen was consumed in the presence of investigated compound (20 μL of 1–2 mM antioxidant solution in acetonitrile was added), in contrast to the reference flask with high concentration (250 μM) of PMHC (**1c**) in order to completely inhibit the oxidation process. Basing on oxygen consumption plots (Δ[O_2_]) with time, for each of the tested compounds, the values of the induction period, τ [s], were graphically determined (linear regression method), as is presented in Figure 5. The values of inhibition rate constant *k*_inh_ [M^−1^s^−1^] and the stoichiometric factor *n* (e.g., the number of peroxyl radicals trapped by a molecule of an antioxidant) were calculated according to the following equations:

Δ[O_2_] = (−*k*_p_/*k*_inh_) × [RH] × ln (1 − t/τ) (1)

*n* = *R*_i_ × τ/[ArOH] (2)

where *k*_p_ is the propagation rate constant [M^−1^s^−1^], [RH] is concentration of oxidizable substrate (styrene/cumene) [M], t is the time [s], *R*_i_ is the rate of initiation [Ms^−1^], and [ArOH] is concentration of the antioxidant.

Rates of chain initiation, *R*_i_ [Ms^−1^], for autoxidation of styrene and cumene in chlorobenzene at 30 °C were determined from preliminary set of experiments from the length of the induction period, using PMHC (**1c**) as a reference antioxidant, for which *n* = 2; thus, transformed Equation (2) can be applied [26]:

*R*_i_ = 2 × [PMHC]/τ (3)

For autoxidation of styrene in chlorobenzene at 30 °C initiated by 0.05 M AIBN, the rate of initiation was determined to be *R*_i_ = 5.53 × 10^−9^ Ms^−1^, while for autoxidation of cumene, it was found to be *R*_i_ = 4.56 × 10^−9^ Ms^−1^ under the same conditions.

### 2.3. DPPH Radicals EPR Measurements

The ability of the studied compounds to reduce the 2,2-diphenyl-1-picrylhydrazyl (DPPH) free radical was performed by electron paramagnetic resonance spectroscopy (EPR) according to the procedure described by Olchowik-Grabarek et al. [27].

**Figure 6:**
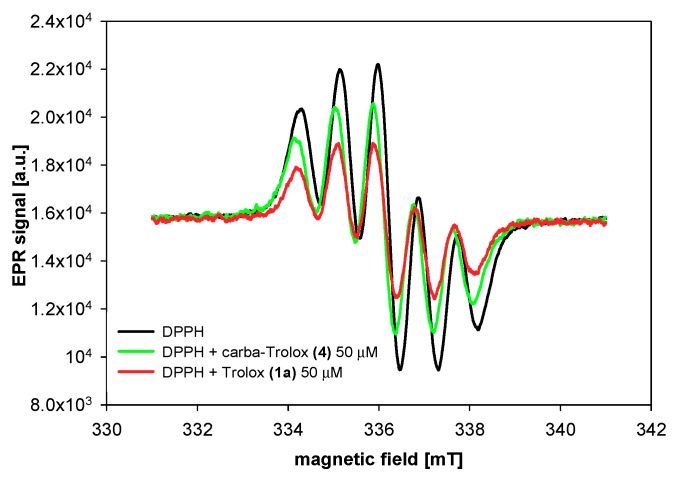
EPR spectra of DPPH radical in the absence (control) and presence of Trolox (**1a**) and carba-Trolox (**4**).

To 500 µM ethanol solution of DPPH● (200 µL), tested compounds were added in different concentrations (1 µL, solution in acetonitrile, final concentration during the measurements: 10–150 µM), mixed, and then transferred into capillaries. The measurements were taken every 2 to 12 min of antioxidant adding. The range of an antioxidant concentration in the reaction system and the frequency of measurements were determined experimentally. EPR spectra were recorded at room temperature (21 ± 1 °C) using an Adani CMS 8400 spectrometer, operating at a microwave frequency of 9.4 GHz. The instrumental settings were as follows: center field 336 mT, sweep width 10 mT, modulation amplitude 0.2 mT, and microwave power 10 dB. The typical EPR spectrum of DPPH● in an organic solvent is presented by five resolved peaks (Figure 6).

### 2.4. Computational Details

The computational data were obtained with the GAUSSIAN 09 suite of programs [28]. To accurately capture properties of the phenolic O–H bond, a B3LYP functional was chosen. The vibrational analysis was performed for each species investigated, confirming its identity as a minimum of energy. Frequencies were determined by using a 6-31G (d,p) basis set to obtain the zero-point energy and the vibrational part of the enthalpy [29]. To account for orbital relaxation upon immersion in solvent, the geometry optimizations were carried out in solutions. Single point calculations were performed by a 6–311++G (2d,2p) basis set to correctly compute the electronic energies. To mimic the solvent environment effect, the polarizable conductor calculation model (CPCM [30]) was used. This theory level, not being computationally demanding, was extensively validated in the past on a number of different problems, like studies on reaction mechanisms [31], equilibrium states [32], and structural analysis of tocopherol-related antioxidants [29]. To account for orbital relaxation upon immersion in solvent, the geometry optimizations were carried out in solutions. 

BDE (bond dissociation enthalpy), IP (ionization potential), PDE (proton dissociation enthalpy), PA (proton affinity), and ETE (electron transfer enthalpy) values were determined from total enthalpies as follows [33]:

BDE = *H*(ArO●) + *H*(H●) − *H*(ArOH) (4)

IP = *H*(ArOH^+^●) + *H*(e^−^) − *H*(ArOH) (5)

PDE = *H*(ArO●) + *H*(H^+^) − *H*(ArOH^+^●) (6)

PA = *H*(ArO^−^) + *H*(H^+^) − *H*(ArOH) (7)

ETE = *H*(ArO●) + *H*(e^−^) − *H*(ArO^−^) (8)

All thermodynamic data were calculated at 298 K. Enthalpies of proton *H*(H^+^) and electron *H*(e^−^) were assumed as 6.1398 and 3.351 kJ/mol, respectively [34,35]. According to the literature, the calculated BDE_OH_ of phenol using the above methodology was 93.1 kcal/mol for the gas phase [36].

## 3. Results and Discussion

### 3.1. Synthesis of all-rac-1-carba-α-tocopherol (**6**)

**Scheme 1:**
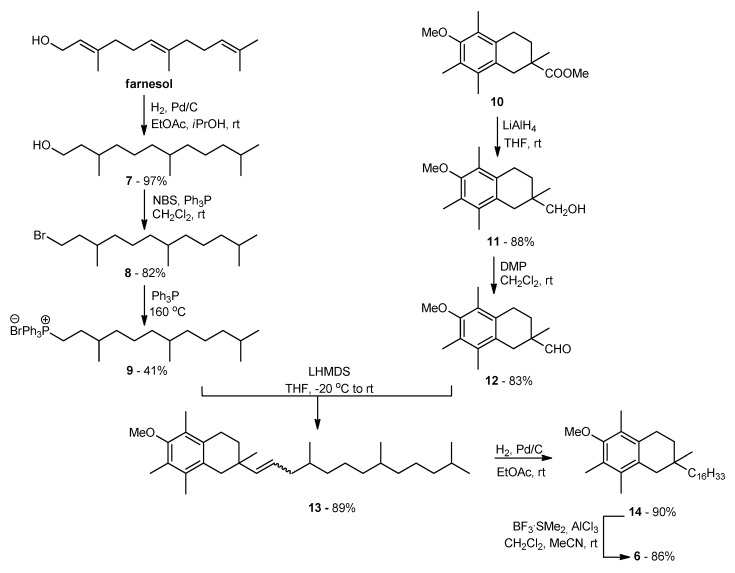
Synthesis of all-*rac*-1-carba-α-tocopherol (**6**).

The main goal of the synthetic work was to obtain vitamin E analogs in which the heterocyclic oxygen atom O1 would be substituted by CH_2_ group. The synthesis of the appropriately substituted 6-hydroxy-1,2,3,4-tetrahydronaphthalene system was described in our previous work [21]. For the synthesis, of the new α-tocopherol analog **6** Wittig reaction was used (Scheme 1). A suitable phosphonium salt was obtained in three steps from commercially available farnesol with 33% overall yield. Farnesol was hydrogenated under atmospheric pressure (10% Pd/C) to give hexahydrofarnesol (**7**), which was converted to bromide **8** using *N*-bromosuccinimide (NBS) in the presence of triphenylphosphine (Ph_3_P). In parallel, compound **10** [21] was converted to the desired aldehyde **12** in two steps: reduction with LiAlH_4_ and then Dess–Martin oxidation. In the next step, aldehyde **12** was coupled with ylide generated in situ from phosphonium salt **9** using LHMDS. The olefin **13** obtained with high yield was subjected to hydrogenation and demethylation successively. All newly prepared compounds were characterized by ^1^H, ^13^C NMR, and FT-IR spectral analysis.

### 3.2. Radical-Scavenging Ability

A number of experimental [5,13,19,20,25,37,38,39,40], as well as theoretical [33,41,42,43,44,45], investigations have been attempted to shed light on the structural factors that determine the high antioxidant and antiradical activity of vitamin E. To quantify the influence of heterocyclic oxygen atom O1, two methods were used. Firstly, the measurement of the rate of autoxidation of model hydrocarbons (styrene and cumene) in the presence of studied compounds was studied. This methodology gives the information about the rate of the reaction with alkyl peroxyl radicals, and therefore yields the ability to quantitatively measure the break up of the peroxidation chains by given compounds. The second technique, a DPPH free radical assay, is a commonly used titration method with an artificial (2,2-diphenyl-1-picrylhydrazyl) radical. This convenient method can give valuable information about the reducing ability of compounds [46]. Thus, measurements of the autoxidation rate are the most reliable methods to gain quantitative information on the antioxidant activity of compounds, because peroxyl radicals are the main mediators of kinetic chain during the propagation step of peroxidation processes.

#### 3.2.1. Measurements of Autoxidation Rate

**Figure 7:**
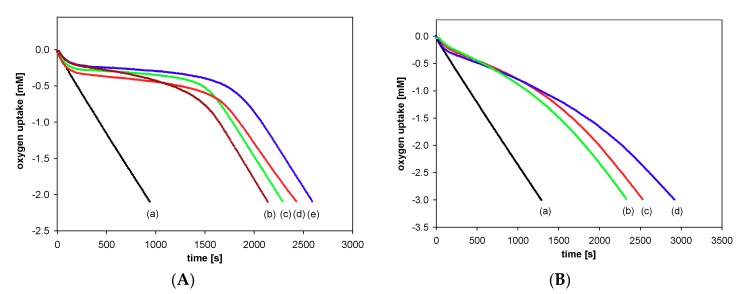
Oxygen uptake during the autoxidation of styrene (4.3 M) in chlorobenzene, initiated by 0.05 M AIBN at 30 °C, in the presence of investigated compounds. (**A**): (**a**) Process uninhibited; (**b**) Trolox (**1a**) (5.0 μM); (**c**) PMHC (**1c**) (5.0 μM); (**d**) Trolox methyl ester (**1b**) (5.1 μM); (**e**) α-tocopherol (**α-T**) (5.1 μM); (**B**): (**a**) Process uninhibited; (**b**) carba-α-tocopherol (**6**) (5.0 μM); (**c**) carba-Trolox (**4**) (5.0 μM); (**d**) carba-Trolox methyl ester (**5**) (7.6 μM).

Antioxidant activity of the synthesized compounds was evaluated based on the rate constant (*k*_inh_) for the reaction with alkyl peroxyl radicals (ROO●) during the controlled inhibited autoxidation of styrene. Figure 7 displays the oxygen consumption during the autoxidation of styrene in the presence of chroman-6-ols (**A**) and the respective 1-carba-analogs (**B**). These plots indicate that the oxygen consumption is strongly inhibited by α-T (blue line) during the initial reaction period, then, when the antioxidant has been completely consumed, the slope of the oxygen uptake trace increases, becoming identical to that observed when the process is uninhibited.

**Figure 8:**
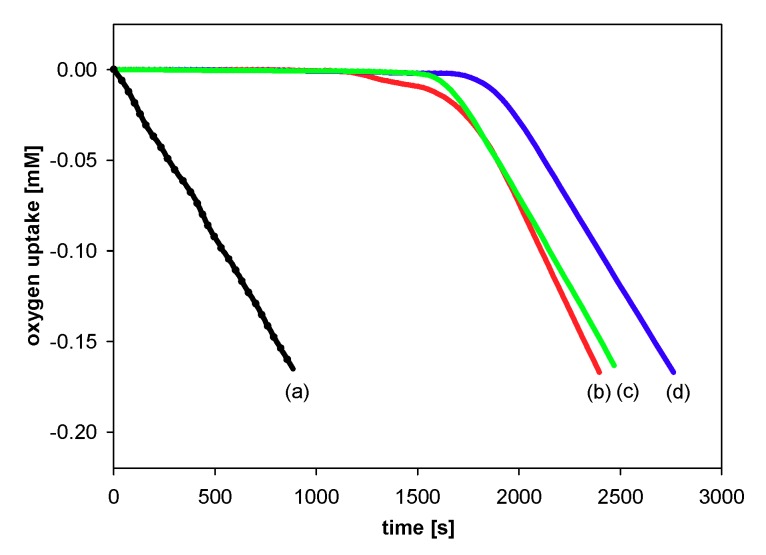
Oxygen uptake during the autoxidation of cumene (3.6 M) in chlorobenzene, initiated by 0.05 M AIBN at 30 °C, in the presence of investigated compounds: (**a**) Process uninhibited; (**b**) carba-α-tocopherol (**6**) (4.0 μM); (**c**) carba-Trolox (**4**) (5.1 μM); (**d**) carba-Trolox methyl ester (**5**) (4.6 μM).

In the case of inhibited autoxidation of styrene in the presence of each of the three 6-hydroxy-1,2,3,4-tetrahydronaphthalenes **4**, **5**, and **6**, the oxygen uptake plots did not show any clear induction periods in comparison to the autoxidation of styrene in the presence of chroman-6-ols used at the same concentrations (Figure 7). This indicates that 1-carba-analogs are much less effective antioxidants than the parent compounds. In order to obtain a more pronounced profile of suppressed oxygen uptake and clear induction periods, the autoxidation of cumene (Figure 8) in the presence of compounds **4**, **5**, and **6** was also investigated. For less efficient antioxidants, characterized by *k*_inh_ 10^−4^–10^−5^ M^−1^s^−1^, cumene is a more suitable oxidizable substrate due to lower (compared to styrene) *k*_p_ value 0.32 M^−1^s^−1^ [47].

The values of calculated inhibition rate constant (*k*_inh_/M^−1^s^−1^), number of peroxyl radicals trapped by one molecule of an antioxidant (stoichiometric factor, *n*), and the length of induction period (τ/s) calculated for the autoxidation of styrene/cumene, inhibited by the investigated antioxidants, are summarized in Table 2. The examination of the obtained results shows that the inhibition rate constants calculated for autoxidation of cumene are very similar to those obtained for carba-analogs **4**–**6** during autoxidation of styrene.

The antioxidant activity of the tested chroman-6-ols decreases in the order: PMHC (**1c**) ≈ α-T > Trolox methyl ester (**1b**) > Trolox (**1a**) according to the literature [5,12]. To quantify the role of O1 atom on the antioxidant activity of vitamin E, the data obtained for 1-carba-analogs were compared with those for the parent system. The *k*_inh_ values found for the carba-analogs **4**, **5**, and **6** were similar to each other, but lower than that of the parent chroman-6-ols (**1a**, **1b**, and α-T). This effect is associated with the removal of stereoelectronic effects coming from the oxygen atom at position 1. Based on the experimental data, it can be concluded that the synthesized 1-carba-analogs are less effective antioxidants, with *k*_inh_ of 10^5^ M^−1^s^−1^ and factor *n* that range from 1.4 to 1.7 (±0.1). The stoichiometric factor should be approximately 2 for efficient phenolic antioxidants. The lowering of *n* value could result from the chain transfer reaction of ArO● with styrene/cumene or self-termination reaction [48]. Replacement of the heterocyclic oxygen atom by CH_2_ group caused a considerable decrease in antioxidant activity: 3-fold for Trolox (**1a**), 5-fold for Trolox methyl ester (**1b**), and 9-fold for α-T.

#### 3.2.2. DPPH Radical Scavenging Activity

DPPH radical scavenging assay gives a general overview of the stoichiometry of the reaction of phenols (or phenol extracts) with model artificial radical and is frequently used as supplementary studies to the kinetic measurements. In our studies, we used EPR detection of DPPH (2,2-diphenyl-1-picrylhydrazyl) radical, but reagents were mixed without the stopped-flow mode and it was not possible to determine the initial reaction rates during the first milliseconds of reaction, as suggested in by Foti [46] and by Amorati and Valgimigli [49] in their critical reviews on DPPH assays. Therefore, our EC_50_ determination gives limited information, typical for titration studies with arbitrarily selected end-point time (here, after 12 min).

**Figure 9:**
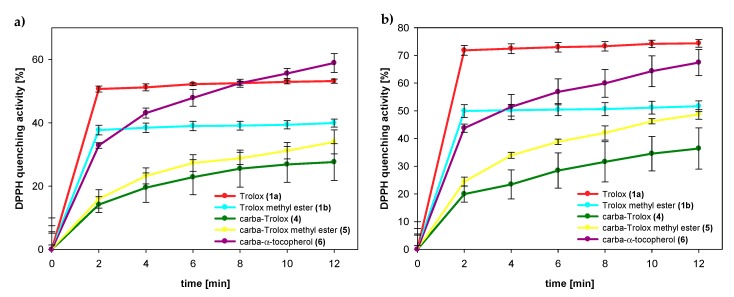
Amount of DPPH radical quenched by tested compounds **1a** (Trolox), **1b** (Trolox methyl ester), **4** (carba-Trolox), **5** (carba-Trolox methyl ester), and **6** (carba-α-tocopherol) in concentrations: (**a**) 50 μM; (**b**) 75 μM.

The amounts of DPPH radicals quenched by the same concentration of selected compounds were monitored during the reaction time. The results obtained 2 and 12 min after initial mixing differed significantly for both series of the compounds: Trolox (**1a**) and its methyl ester (**1b**) from one side and the respective 1-carba-analogs from another side. As can be seen in Figure 9 and Table 3, Trolox (**1a**; 75 µM) had the highest reducing ability, for which we observed 72% reduction of the initial amount of DPPH● after 2 min and almost the same conversion (74%) after 12 min. Trolox methyl ester (**1b**) was less active than Trolox (**1a**), and after 2 min, the percentage radicals neutralized by **1b** (75 µM) was 50% (plateau state). In contrast, the acid **4** (75 µM) reacted a little slower than ester **5**. After 2 min, the amount of scavenged DPPH radicals by **4** was 20% and increased to 36% in the next 10 min.

In the case of all 1-carba-analogs, plateau state was not reached within 12 min, which proves a much slower reaction with DPPH radical compared to the parent compounds with oxygen atom in para-position to the phenolic OH group. However, after 12 min, the amount of radical quenched by the carbocyclic analog of Trolox methyl ester (**5**) was 49%, which is almost the same as for parent **1b**.

The calculated EC_50_ values for Trolox (**1a**) and 1-carba-Trolox (**4**) are 48 µM and 107 µM, respectively (Table 3). A smaller difference (69 µM and 75 µM) was observed for Trolox methyl ester (**1b**) and carbocyclic analog **5**. Nevertheless, these results confirm that 1-carba-analogs **4**, **5**, and **6** are much less effective scavenging agents than the parent chroman-6-ols. We can exclude the possibility of the deprotonation of the OH group in ethanol and sequential proton loss–electron transfer [40], as Trolox (**1a**) and 1-carba-Trolox (**4**) have carboxyl groups being stronger acids than the phenolic hydroxyl group. Thus, any deprotonation of the OH group is shifted back. Supposedly, the observed difference in reactivity of the analogs must result from a different strength of O–H bond (higher BDE_OH_ value).

### 3.3. DFT Calculations

The purpose of our research was to compare the antioxidant activity of chroman-6-ols and the corresponding 1-carba-analogs. The experimental results were supported by DFT calculations in the gas phase and the presence of selected solvents. In this paper, three mechanisms of antioxidant action were considered: HAT (hydrogen atom transfer), SET–PT (sequential electron transfer—proton transfer), and SPLET (sequential proton loss—electron transfer) [50]. Generally, in apolar solvents, the HAT mechanism is predominant, but in polar solvents (e.g., methanol, ethanol), the SET–PT mechanism becomes favorable because of its ability to form strong hydrogen bonds with an ArOH molecule. The BDE (bond dissociation enthalpy), IP (ionization potential), PDE (proton dissociation enthalpy), PA (proton affinity), and ETE (electron transfer enthalpy) values were calculated for the tested compounds in various environments (Table 4). HAT is a one-step mechanism (Equation (9)), while SET–PT (Equations (10) and (11)) and SPLET (Equations (12) and (13)) run in two steps and involve the formation of an intermediate [43]. Hence, the present study mainly focused on the BDE, IP, and PA, characterized by the first steps in three mechanisms. In the HAT and SPLET mechanisms, the kinetics of the reaction of phenols with electron-deficient radicals are characterized by the lowest BDE and lowest PA values. BDE is a representative factor in the determination of the efficacy of a phenolic antioxidant. The lower the BDE value, the weaker the O–H bond and the faster the reaction of an antioxidant with radicals. Ionization potential (IP) allows the evaluation of the ability of phenol to donate electrons.

**HAT:** ArOH●→ ArO● + H● (9)

**SET–PT:** ArOH●→ ArOH^+^● + e**^−^** (10)

ArOH^+^● → ArO● + H**^+^** (11)

**SPLET:** ArOH●→ ArO**^−^** + H**^+^** (12)

ArO**^−^**→ ArO● + e^−^ (13)

The calculated BDE, IP, and PA values were lower in ethanol than those in chlorobenzene and gas phase. The lowest BDE and IP values in all environments were observed for α-T. In the chroman-6-ols, the BDE value decreases in the order: **1a** > **1b** > α-T in all environments. Within the series of 1-carba-analogs, the calculated BDE values did not differ significantly. The difference between BDE values for the 6-hydroxy-tetralins and the parent chromanols ranged from 2.9 to 4.2 kcal/mol. For example, in the gas phase, ΔBDE for pairs: **4**/**1a**, **5**/**1b**, and **6**/α-T were ca. 2.9, 3.2, and 3.7 kcal/mol, respectively. Similarly, 1-carba-analogs had higher IP values than chromanols**.** The difference between IP values for 1-carba-α-T (**6**) and α-T was ca. 11.4, 9.5, and 9.0 kcal/mol in the gas phase, chlorobenzene, and ethanol, respectively. The differences between PA for chromanols and tetralols were rather small; however, in this case, 1-carba-analogs had lower PA values. The gas phase difference for pairs: **4**/**1a**, **5**/**1b**, and **6**/α-T were −1.7, −2.7, and −2.1 kcal/mol, respectively. In conclusion, in gas phase, chlorobenzene, and ethanol, BDE values are lower than PA and IP values for all tested compounds, which means HAT represents the most probable pathway. We can extend this conclusion to lipid bilayers, because tocopherols, as lipid-soluble antioxidants, are located in such non-polar environments. However, even in lipid bilayers, the rate of hydrogen atom transfer from phenols to peroxyl radicals can be slower due to hydrogen bonding of phenolic hydroxy group with some functional groups in the polar part of lipid bilayer [52].

## 4. Conclusions

The high antioxidant activity of α-tocopherol (α-T) is mainly attributed to stereoelectronic effects exerted by oxygen atom in dihydropyranyl ring. The orbital overlap between the 2p-type lone pair on the heterocyclic oxygen atom (O1) and the aromatic π-electron system stabilizes chromanyloxyl radicals formed. Quantitative estimation of the influence of the O1 atom on the antioxidant activity of chroman-6-ols was attempted. For this purpose, several derivatives of 6-hydroxy-1,2,3,4-tetrahydronaphthalene as model 1-carba-analogs of the chroman-6-ol system were synthesized. In our opinion, analogs deprived of the O1 atom (substituted by CH_2_) are more structurally related to the parent compounds and enable more precise estimation of the contribution of the O1 atom for stabilization of the respective phenoxyl radicals.

In this paper we described the first synthesis of carbocyclic analog of α-tocopherol (1-carba-α-T). The model 6-hydroxy-1,2,3,4-tetrahydronaphthalenes were tested in vitro with regard to the antioxidant activity. The inhibition of controlled initiation of model hydrocarbon (styrene and cumene) oxidation and 2,2-diphenyl-1-picrylhydrazyl (DPPH) free radical quenching assay were used. Our results are consistent with the Burton and Ingold concept, whereby the high antioxidant activity of vitamin E is attributed to the stereoelectronic effects of the O1 atom in the dihydropyranyl ring of the chroman-6-ol system. The results of these authors were based on measurements of antioxidant activity in comparison to much simpler model compounds, pentamethylphenol and 4-methoxy-2,3,5,6-tetramethylphenol, which differ significantly from the structure of the parent α-tocopherol. The use of model 1-carba-analogs for this purpose is a new approach.

The influence of the oxygen atom O1 on the antioxidant activity of α-T (vitamin E) was also theoretically calculated using the DFT method. The BDE, IP, PDE, PA, and ETE parameters were determined in the gas phase and in the presence of selected solvents. Based on the optimized geometries, reaction enthalpies related to the HAT, SET–PT, and SPLET mechanisms were discussed.

## Figures and Tables

**Table 1:** Rate constant *k*_inh_ for selected phenolic antioxidants [5].

Structure	Phenol	10^5^ *k*_inh_ [M^−1^ s^−1^]
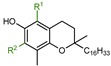	**α-T**: R^1^ = CH_3_, R^2^ = CH_3_	32.0
	**β-T**: R^1^ = CH_3_, R^2^ = H	13.0
	**γ-T**: R^1^ = H, R^2^ = CH_3_	14.0
	**δ-T**: R^1^ = H, R^2^ = H	4.4
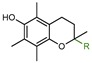	**1a**: R = COOH (Trolox)	11.0
	**1b**: R = COOCH_3_ (Trolox methyl ester)	18.0
	**1c**: R = CH_3_ (PMHC)	38.0
	**1d**: R = (CH_2_)_2_COOH	37.0
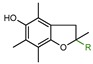	**2a**: R = H	54.0
	**2b**: R = CH_3_	57.0
	**2c:** R = COOH	16.0
	**2d**: R = C_16_H_33_ [18]	47.0
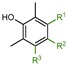	**3a**: R^1^ = H, R^2^ = OCH_3_, R^3^ = CH_3_	13.0
	**3b**: R^1^ = H, R^2^ = OCH_3_, R^3^ = H	9.4
	**3c**: R^1^ = CH_3_, R^2^ = OCH_3_, R^3^ = CH_3_	3.9
	**3d**: R^1^ = CH_3_, R^2^ = CH_3_, R^3^ = CH_3_	3.6

Values *k*_inh_ were measured by the inhibited autoxidation of styrene at 30 °C. α-T, α-Tocopherol; β-T, β-Tocopherol; γ-T, γ-Tocopherol; δ-T, δ-Tocopherol.

**Table 2:** The values of inhibition rate constant (*k*_inh_/M^−1^s^−1^) and the number of peroxyl radicals trapped by one molecule of antioxidant (*n*) calculated for the autoxidation of styrene/cumene in chlorobenzene at 30 °C, inhibited by investigated compounds *.

Phenol	*k*_inh_ [M^−1^s^−1^]	*n* **
	**Autoxidation of Styrene**	
**1a**	(9.0 ± 0.4) × 10^5^	1.6
**1b**	(1.5 ± 0.1) × 10^6^	1.7
**α-T**	(2.0 ± 0.2) × 10^6^	1.9
**1c**	(2.2 ± 0.1) × 10^6^	1.9
**4**	(2.8 ± 0.1) × 10^5^	1.6
**5**	(3.1 ± 0.2) × 10^5^	1.4
**6**	(2.3 ± 0.1) × 10^5^	1.5
	**Autoxidation of Cumene**	
**4**	(2.6 ± 0.4) × 10^5^	1.5
**5**	(3.1 ± 0.2) × 10^5^	1.7
**6**	(2.3 ± 0.5) × 10^5^	1.5

* Results are presented as means ± standard deviation (SD) from at least three independent experiments. Full data, including the concentrations of antioxidants and induction periods determined for each experiment, are included in Supplementary File S1. ** The value of SD for each stoichiometric factor (***n***) determined is ± 0.1.

**Table 3:** EC_50_ and ARP values of tested compounds *.

Compound	EC_50_ [µM]	ARP [1/EC_50_ × 10^−3^]
**1a**	48.0 ± 0.1	20.8 ± 0.1
**1b**	68.9 ± 2.3	14.5 ± 0.5
**4**	106.8 ± 2.1	9.4 ± 0.2
**5**	75.1 ± 3.3	13.3 ± 0.6
**6**	46.8 ± 3.7	21.5 ± 1.7

* EC_50_ value was defined as the concentration required causing 50% of maximum effect. ARP, antiradical power. Results are expressed as means ± SD from at least four independent experiments.

**Table 4:** The BDE, IP, PDE, PA, and ETE (in kcal/mol) calculated values for investigated antioxidants in different environments.

Environment	Compound	BDE	IP	PDE	PA	ETE
**Gas phase**	**1a**	78.3	161.6	231.2	350.2	39.9
	**1b**	78.1	161.2	231.4	350.9	40.0
	**α-T**	77.3*	157.2	234.5	352.2	38.2
	**4**	81.2	171.2	224.6	348.5	45.3
	**5**	81.3	171.6	224.2	348.2	45.4
	**6**	81.0	168.6	226.9	350.1	43.4
**Chlorobenzene**	**1a**	77.2	132.2	259.4	311.6	77.3
	**1b**	77.1	132.0	259.6	311.6	78.1
	**α-T**	75.9	128.6	261.8	312.8	75.8
	**4**	80.3	139.8	255.0	310.7	81.9
	**5**	80.4	139.9	255.0	310.7	82.0
	**6**	79.9	138.1	256.3	311.6	80.7
**Ethanol**	**1a**	76.9	127.4	263.9	304.6	84.0
	**1b**	76.8	127.4	263.9	304.6	84.7
	**α-T**	75.4	124.6	265.3	305.5	82.7
	**4**	80.1	134.6	260.0	303.9	88.4
	**5**	80.1	135.0	259.7	303.9	88.5
	**6**	79.6	133.6	260.4	304.5	87.4

*BDE_OH_ ≈ 77 kcal/mol [7,25,51]. BDE, bond dissociation enthalpy; IP, ionization potential; PDE, proton dissociation enthalpy; PA, proton affinity; ETE, electron transfer enthalpy.

## Supplementary Materials

The following data are available online at https://www.mdpi.com/2076-3921/8/12/589/s1: Supplementary File S1: Experimental details for synthesis of phosphonium salt **9**; NMR spectra for all intermediates and the final product **6**; styrene/cumene autoxidation measurements.

## Abbreviations

ARP	antiradical power
BDE	bond dissociation enthalpy
DFT	density functional theory
DPPH●	2,2-diphenyl-1-picrylhydrazyl free radical
EC_50_	the concentration required causing 50% of maximum effect
ETE	electron transfer enthalpy
HAT	hydrogen atom transfer
IP	ionization potential
LOO●	lipid peroxyl radical
PA	proton affinity
PDE	proton dissociation enthalpy
PMHC	2,2,5,7,8-pentamethylchroman-6-ol
ROO●	alkyl peroxyl radical
SET-PT	sequential electron transfer—proton transfer
SPLET	sequential proton loss—electron transfer
Trolox	6-hydroxy-2,5,7,8-tetramethylchromane-2-carboxylic acid
