# Supplementary material for: Synthesis, DFT Calculations, and In Vitro Antioxidant Study on Novel Carba-Analogs of Vitamin E
Source: Antioxidants (Basel). 2019 Nov 26;8(12):589. doi: 10.3390/antiox8120589 (PMC6943657; doi:10.3390/antiox8120589)
Supplement: Supplementary file 1 [file antioxidants-08-00589-s001.pdf]

SUPPORTING INFORMATION

for

Synthesis, DFT Calculations and In Vitro Antioxidant Study on Novel Carba-Analogs of Vitamin E

Aneta Baj [1], Jakub Cedrowski [2], Ewa Olchowik-Grabarek [3], Artur Ratkiewicz [1] and Stanislaw Witkowski [1,*]

[1] Faculty of Chemistry, University of Bialystok, Ciolkowskiego 1K, 15-245 Bialystok, Poland
[2] Faculty of Chemistry, University of Warsaw, Pasteura 1, 02-093 Warsaw, Poland
[3] Faculty of Biology, University of Bialystok, Ciolkowskiego 1J, 15-245 Bialystok, Poland
* Correspondence: wit@uwb.edu.pl (S.W.)

Table of Contents

Synthesis of phosphonium salt **9** S2

Styrene autoxidation inhibited by compounds **1a**, **1b**, **α-T**, **1c**, **4**, **5** and **6** (Table 1S) S3

Cumene autoxidation inhibited by compounds **4**, **5** and **6** (Table 2S) S4

^{1}H and ^{13}C NMR spectra of compound **6** S5-S6

^{1}H and ^{13}C NMR spectra of compound **7** S7-S8

^{1}H and ^{13}C NMR spectra of compound **8** S9-S10

^{1}H and ^{31}P NMR spectra of compound **9** S11-S12

^{1}H and ^{13}C NMR spectra of compound **11** S13-S14

^{1}H and ^{13}C NMR spectra of compound **12** S15-S16

^{1}H NMR spectrum of compound **13** S17

^{1}H NMR spectrum of compound **14** S18

Synthesis of phosphonium salt **9**

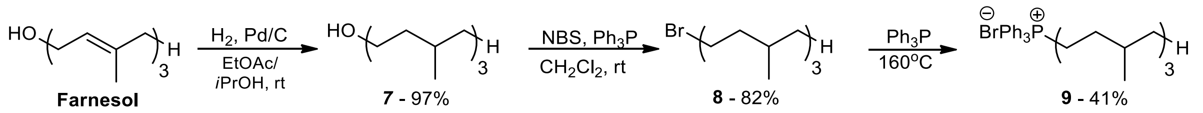

3,7,11-Trimethyldodecyl alcohol (**7**).

A mixture of farnesol (3 g, 13.5 mmol) and 10% Pd/C (0.3 g) in ethyl acetate (15 mL) and propan-2-ol (15 mL) was stirred and hydrogenated under atmospheric pressure at room temperature. After 24 h the reaction mixture was filtered through a pad of Celite and concentrated under reduced pressure. Yellow oily residue was purified by DFC (hexane/ethyl acetate, 8:2, v/v) to give compound **7** as a colorless oil (2.98 g, 97%). R_f = 0.43 (hexane/ethyl acetate, 8:2, v/v); ^{1}H NMR (400 MHz, $CDCl_3$): δ 3.71-3.66 (m, 2H, C<u>H</u>$_2$OH), 1.63-1.49 (m, 3H), 1.39-1.04 (m, 15H), 0.90 (d, J = 6.5 Hz, 3H), 0.87 (d, J = 6.6 Hz, 6H), 0.85 (d, J = 6.8 Hz, 3H) ppm; ^{13}C NMR (100 MHz, $CDCl_3$): δ 61.23, 40.03, 39.95, 39.34, 37.48, 37.44, 37.35, 37.31, 37.25, 32.77, 32.76, 29.51, 29.49, 27.96, 24.79, 24.77, 24.35, 22.70, 22.60, 19.72, 19.66, 19.64, 19.60 ppm; IR (ATR): 3336, 2924, 1459, 1054 cm^{-1}.

3,7,11-Trimethyldodecyl bromide (**8**).

Compund **8** was prepared according to the method proposed by Sagara et al. with minor modifications [1]. NBS (2.705 g, 15.2 mmol) was added portionwise to a solution of compound **7** (2.9 g, 12.7 mmol) and Ph_3P (3.672 g, 14.0 mmol) in dry CH_2Cl_2 (50 mL) under argon at 0°C. After stirring for 18 h at room temperature solvent was removed with a rotary evaporator. The residue was dispersed in hexane and purified by FC (hexane as eluent) to obtain bromide **8** as a colorless oil (3.05 g, 82%). R_f = 0.91 (hexane). ^{1}H NMR (400 MHz, $CDCl_3$): δ 3.51-3.38 (m, 2H, C<u>H</u>$_2$Br), 1.90-1.87 (m, 1H), 1.71-1.64 (m, 2H), 1.57-1.50 (m, 1H), 1.48-1.02 (m, 13H), 0.90 (d, J = 6.2 Hz, 3H), 0.88 (d, J = 6.7 Hz, 6H), 0.85 (d, J = 6.4 Hz, 3H) ppm; ^{13}C NMR (100 MHz, $CDCl_3$): δ 40.10, 40.03, 39.34, 37.35, 37.27, 37.24, 37.21, 36.83, 36.76, 32.75, 32.73, 32.22, 31.67, 31.63, 27.97, 24.80, 24.79, 24.21, 22.71, 22.62, 19.72, 19.65, 18.99, 18.93 ppm; IR (ATR): 2924, 1459, 1378, 1259, 614 cm^{-1}.

Triphenyl(3,7,11-trimethyldodecyl)phosphonium bromide (**9**).

Mixture of bromide **8** (2.75 g, 9.4 mmol) and Ph_3P (2.473 g, 9.4 mmol) was stirred vigorously at 160°C for 24 h. After cooling to room temperature, Et_2O was added and concentrated under reduced pressure. White solid was triturated with cold ether, filtered, washed with cold ether and dried under high vacuum (80°C, 24 h) to give product **9** as a white solid (2.132 g, yield 41%). ^{31}P NMR (161 MHz, $CDCl_3$): δ 25.26 ppm; ^{1}H NMR (400 MHz, $CDCl_3$): δ 7.86-7.42 (m, 15H), 3.71-3.65 (m, 2H), 2.03 (brs, 1H), 1.77-1.75 (m, 1H), 1.60-1.52 (m, 1H), 1.51-1.47 (m, 1H), 1.46-1.40 (m, 1H), 1.40-0.99 (m, 12H), 0.97 (d, J = 6.6 Hz, 3H), 0.84 (d, J = 6.6 Hz, 6H), 0.77 (d, J = 6.6 Hz, 3H) ppm; ^{13}C NMR (100 MHz, $CDCl_3$): δ 135.00, 134.97, 133.71, 133.61, 130.52, 130.40, 118.73, 117.88, 39.27, 37.26, 37.18, 37.17, 37.13, 36.66, 33.59, 33.56, 33.44, 33.42, 32.70, 29.43, 29.38, 29.34, 29.29, 27.89, 24.73, 24.70, 24.17, 24.13, 22.66, 22.56, 21.17, 20.67, 19.63, 19.51, 19.21, 19.15 ppm; IR (KBr): 2934, 1530, 1404, 1104, 701 cm^{-1}; MS (ESI+): m/z = 473.1 ([M-Br]$^+$).

1. Sagara, Y.; Kato, T. Brightly Tricolored Mechanochromic Luminescence from a Single-Luminophore Liquid Crystal: Reversible Writing and Erasing of Images. *Angew. Chem. Int. Ed.* **2011**, *50*, 9128–9132.

Table 1S. The values of inhibition rate constant (k_{inh} / $M^{-1}s^{-1}$), number of peroxyl radicals trapped by one molecule of antioxidant (n) and the length of induction period (τ / s) calculated for autoxidation of styrene in chlorobenzene at 30°C, inhibited by compounds **1a**, **1b**, **α-T**, **1c**, **4**, **5** and **6**.

Structure of phenol	No.	k_{inh} [$M^{-1}s^{-1}$]	n	τ [s]	ArOH [μM]
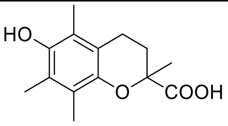	1	9.06×10^5	1.60	1450	5.02
	2	9.41×10^5	1.55	1404	5.02
	3	8.56×10^5	1.77	1610	5.02
**1a**		**$(9.01\pm0.43)\times10^5$**	**1.64±0.12**		
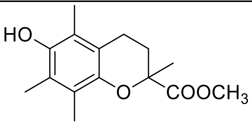	1	1.35×10^6	1.61	1489	5.10
	2	1.54×10^6	1.73	1594	5.10
	3	1.50×10^6	1.74	1607	5.10
**1b**		**$(1.46\pm0.10)\times10^6$**	**1.69±0.07**		
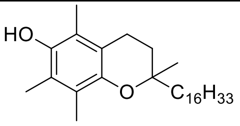	1	1.87×10^6	1.94	1778	5.07
	2	2.27×10^6	1.87	1712	5.07
	3	1.91×10^6	1.89	1729	5.07
**α-T**		**$(2.02\pm0.22)\times10^6$**	**1.90±0.04**		
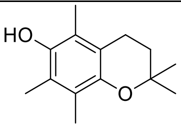	1	2.15×10^6	1.92	1732	4.99
	2	2.33×10^6	1.84	1657	4.99
	3	2.26×10^6	1.90	1717	4.99
**1c**		**$(2.24\pm0.09)\times10^6$**	**1.89±0.04**		
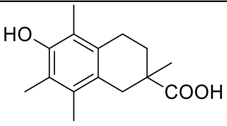	1	2.83×10^5	1.51	1452	5.33
	2	2.75×10^5	1.48	1342	5.01
	3	2.88×10^5	1.66	1508	5.01
**4**		**$(2.82\pm0.07)\times10^5$**	**1.55±0.10**		
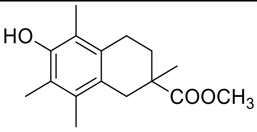	1	3.06×10^5	1.53	1051	3.80
	2	3.31×10^5	1.30	1781	7.56
	3	2.88×10^5	1.31	1796	7.56
**5**		**$(3.08\pm0.22)\times10^5$**	**1.38±0.13**		
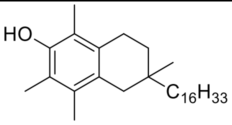	1	2.92×10^5	1.55	1371	4.03
	2	2.02×10^5	1.46	863	2.69
	3	2.09×10^5	1.60	944	2.69
**6**		**$(2.34\pm0.50)\times10^5$**	**1.54±0.07**		

Table 2S. The values of inhibition rate constant (k_{inh} / $M^{-1}s^{-1}$), number of peroxyl radicals trapped by one molecule of antioxidant (n) and the length of induction period (τ / s) calculated for autoxidation of cumene in chlorobenzene at 30°C, inhibited by compounds **4**, **5** and **6**.

**Structure of phenol**	**No.**	k_{inh} [$M^{-1}s^{-1}$]	n	**τ [s]**	**ArOH [μM]**
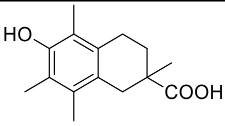	1	2.74×10^{5}	1.50	1258	3.81
	2	2.77×10^{5}	1.58	1764	5.08
	3	2.14×10^{5}	1.53	1711	5.08
**4**		**(2.55±0.36)×10^5**	**1.54±0.04**		
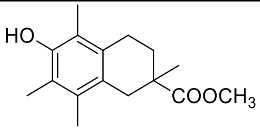	1	2.95×10^{5}	1.59	1614	4.64
	2	3.26×10^{5}	1.75	1779	4.64
	3	3.11×10^{5}	1.77	1804	4.64
**5**		**(3.11±0.16)×10^5**	**1.70±0.10**		
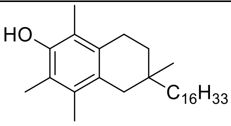	1	2.92×10^{5}	1.55	1371	4.03
	2	2.02×10^{5}	1.46	863	2.69
	3	2.09×10^{5}	1.60	944	2.69
**6**		**(2.34±0.50)×10^5**	**1.54±0.07**		

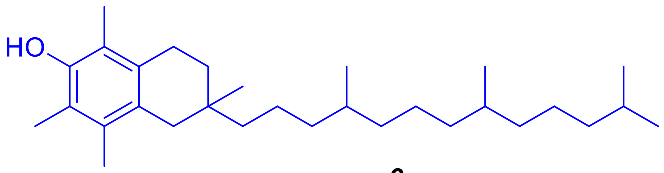
HO

6

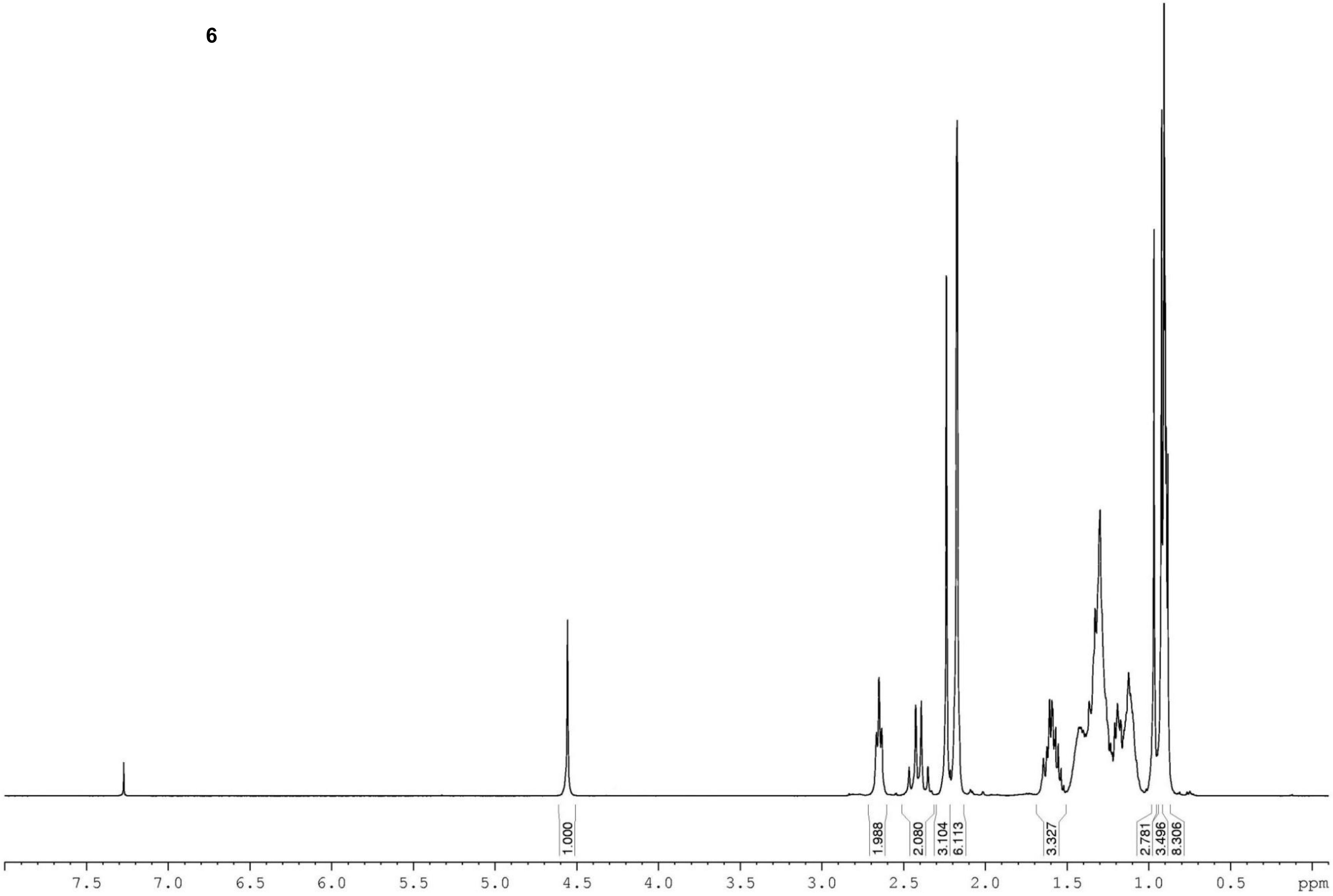
1.000
1.988
2.080
3.104
6.113
3.327
2.781
3.496
8.306
7.5
7.0
6.5
6.0
5.5
5.0
4.5
4.0
3.5
3.0
2.5
2.0
1.5
1.0
0.5
ppm

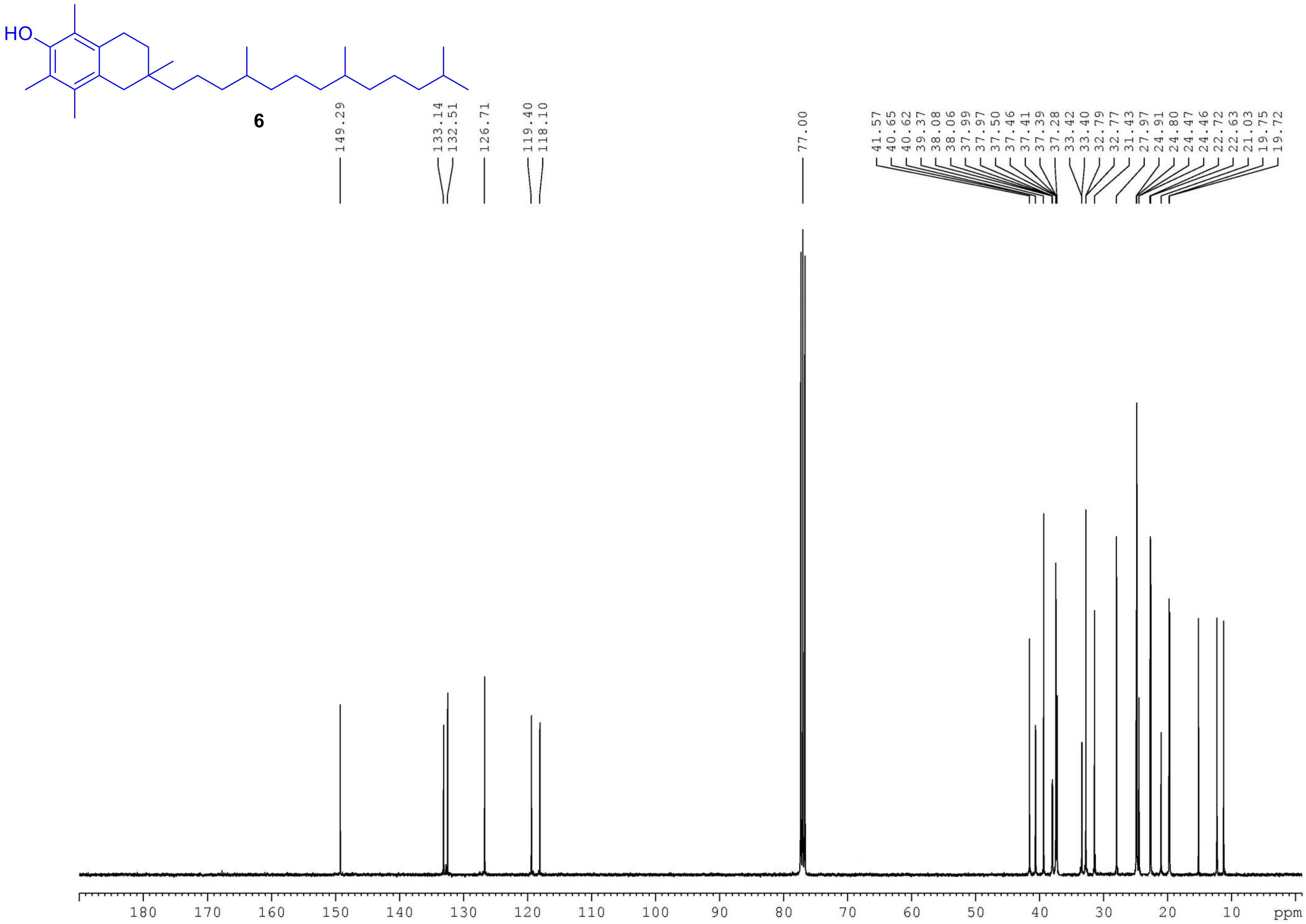
HO
6
149.29
133.14
132.51
126.71
119.40
118.10
77.00
41.57
40.65
40.62
39.37
38.08
38.06
37.99
37.97
37.50
37.46
37.41
37.39
37.28
33.42
33.40
32.79
32.77
31.43
27.97
24.91
24.80
24.47
24.46
22.72
22.63
21.03
19.75
19.72
180 170 160 150 140 130 120 110 100 90 80 70 60 50 40 30 20 10 ppm

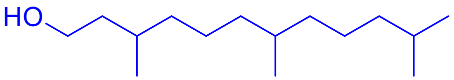
HO

7

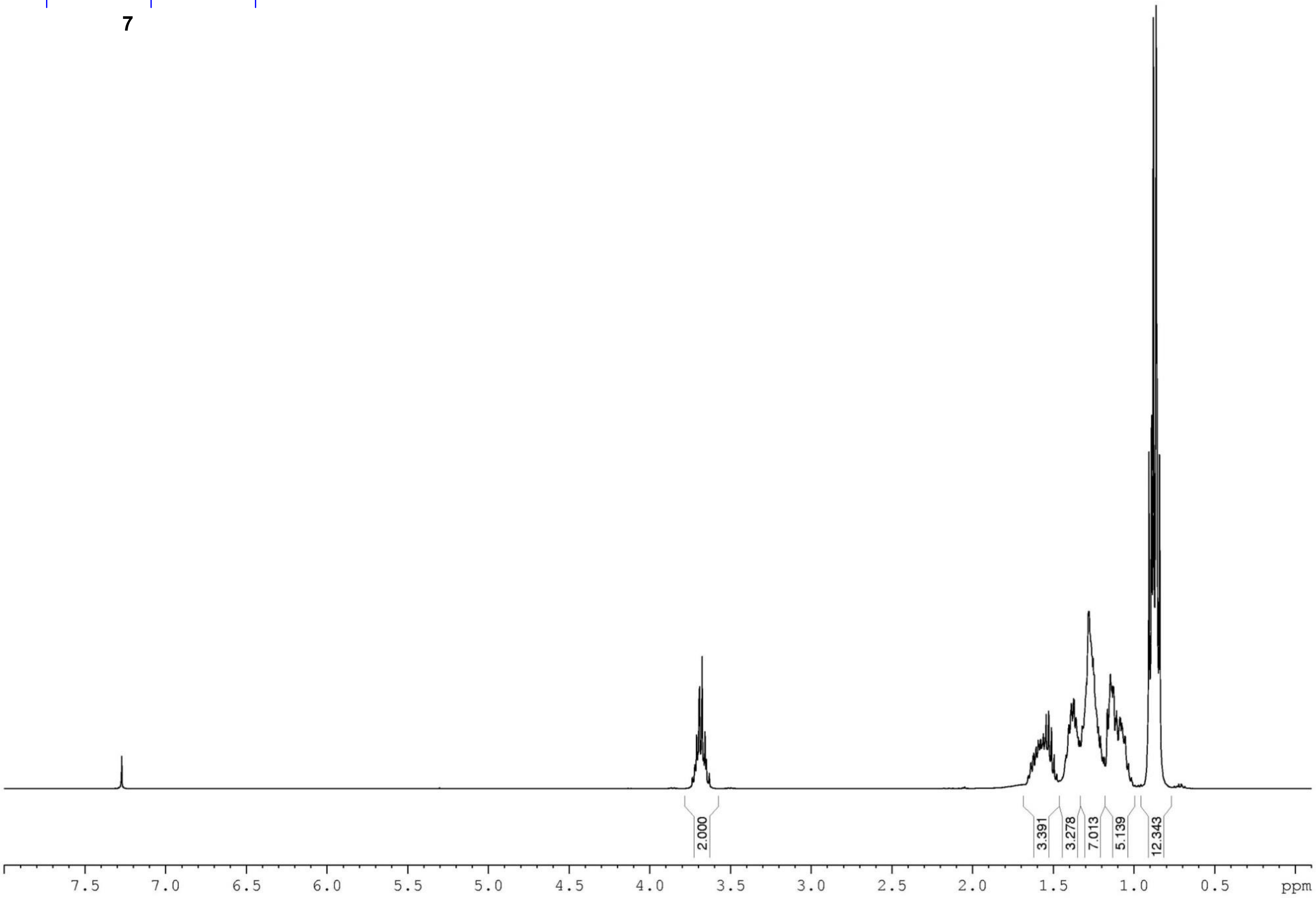
2.000
3.391
3.278
7.013
5.139
12.343
7.5
7.0
6.5
6.0
5.5
5.0
4.5
4.0
3.5
3.0
2.5
2.0
1.5
1.0
0.5
ppm

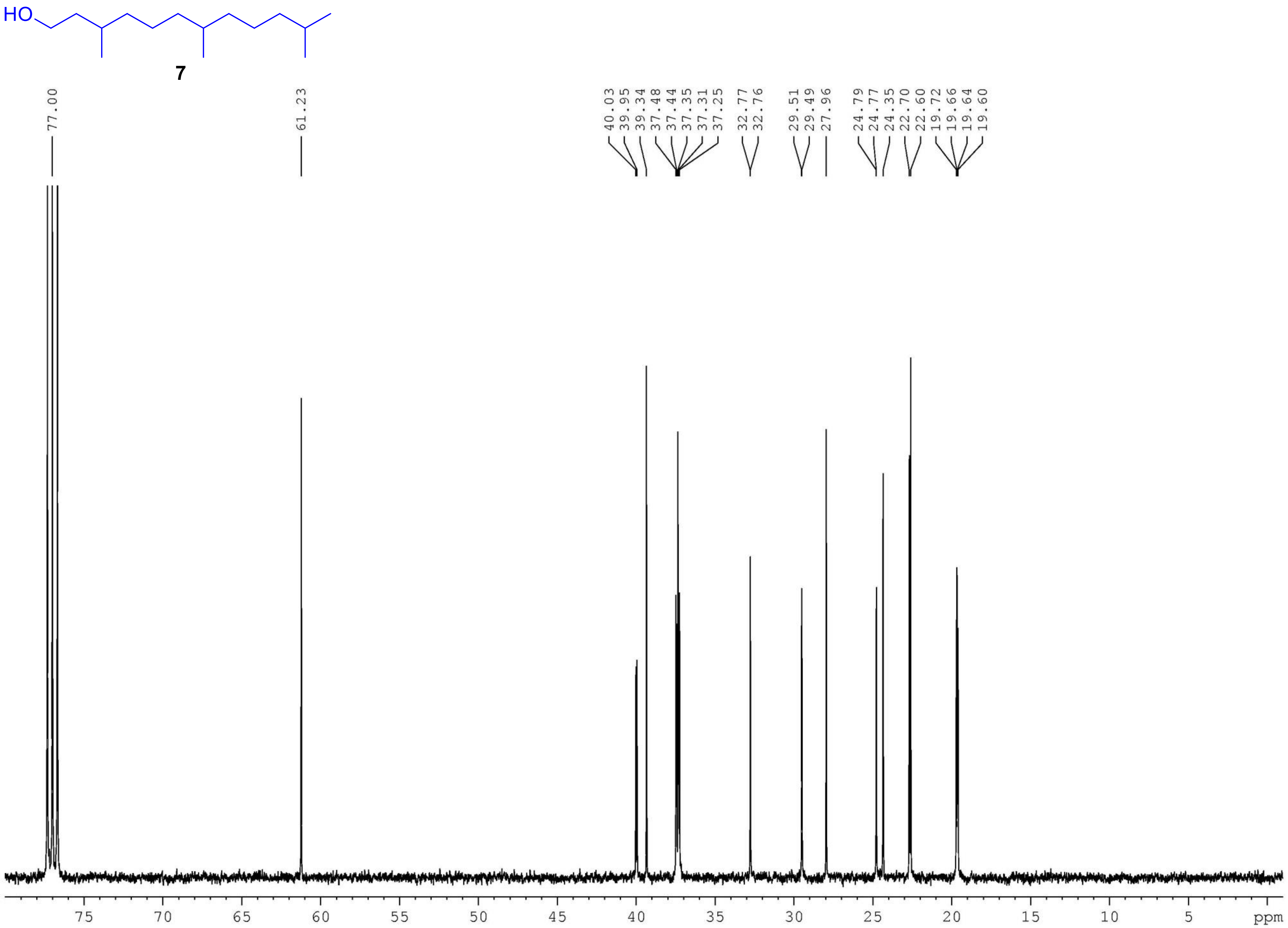
HO
7
77.00
61.23
40.03
39.95
39.34
37.48
37.44
37.35
37.31
37.25
32.77
32.76
29.51
29.49
27.96
24.79
24.77
24.35
22.70
22.60
19.72
19.66
19.64
19.60
75
70
65
60
55
50
45
40
35
30
25
20
15
10
5
ppm

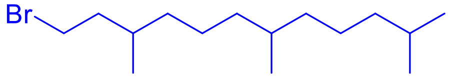

8

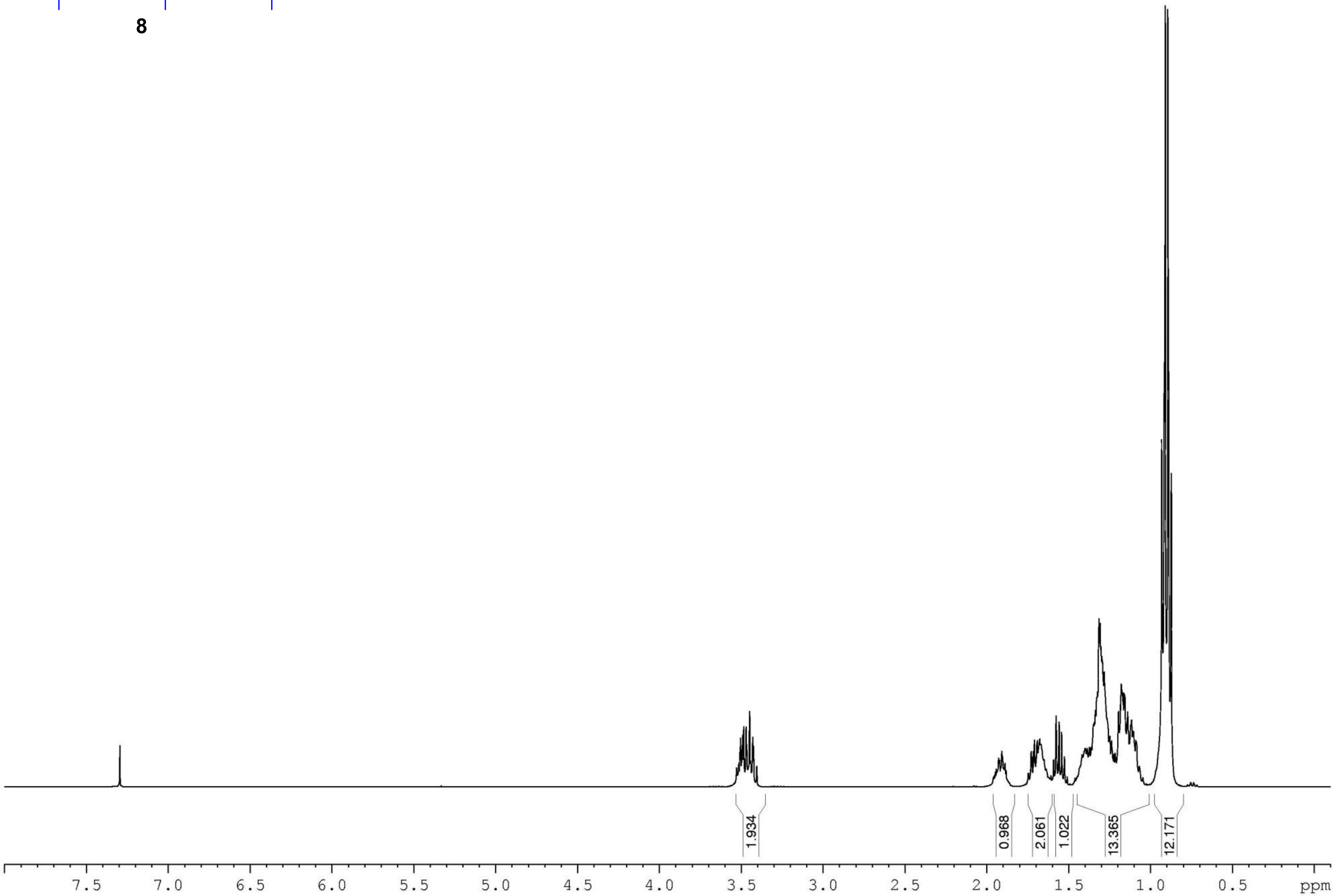
1.934
0.968
2.061
1.022
13.365
12.171
7.5
7.0
6.5
6.0
5.5
5.0
4.5
4.0
3.5
3.0
2.5
2.0
1.5
1.0
0.5
ppm

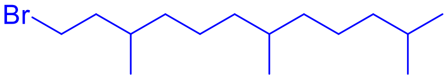
Br

8

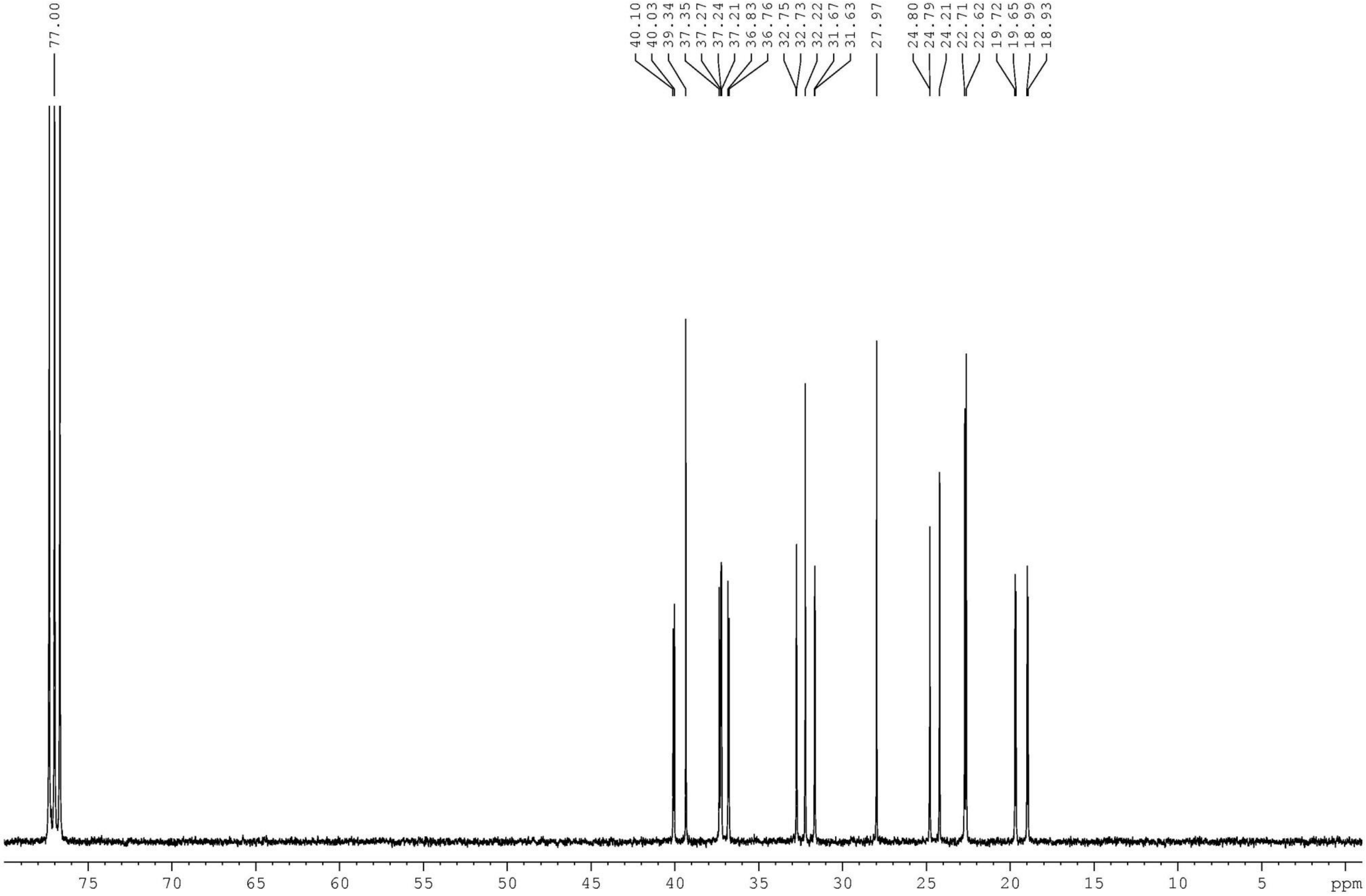
77.00
40.10
40.03
39.34
37.35
37.27
37.24
37.21
36.83
36.76
32.75
32.73
32.22
31.67
31.63
27.97
24.80
24.79
24.21
22.71
22.62
19.72
19.65
18.99
18.93
75
70
65
60
55
50
45
40
35
30
25
20
15
10
5
ppm

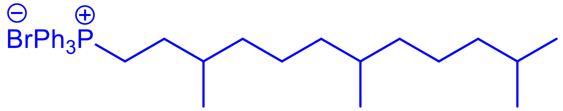
BrPh3P

9

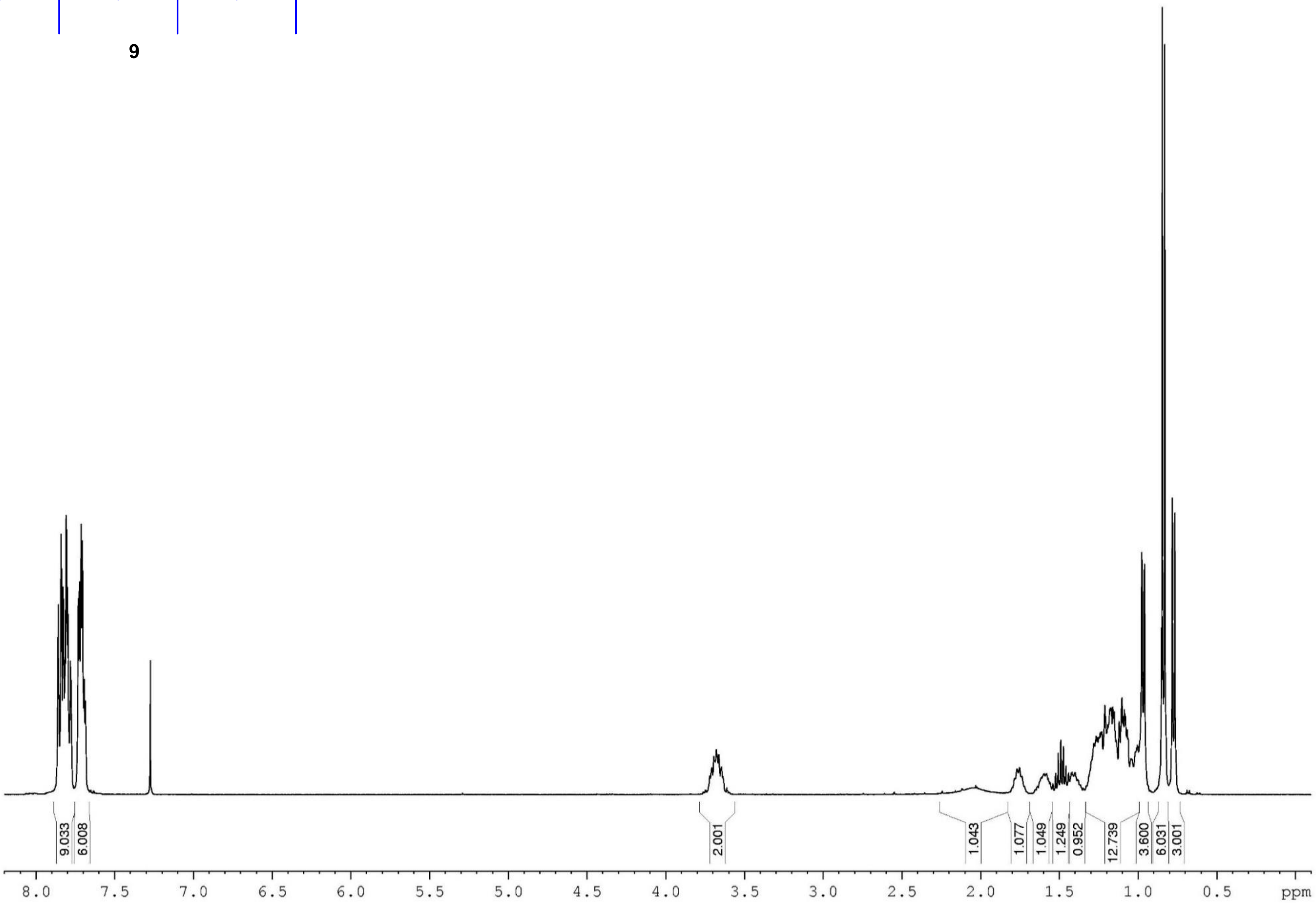
9.033
6.008
2.001
1.043
1.077
1.049
1.249
0.952
12.739
3.600
6.031
3.001
8.0
7.5
7.0
6.5
6.0
5.5
5.0
4.5
4.0
3.5
3.0
2.5
2.0
1.5
1.0
0.5
ppm

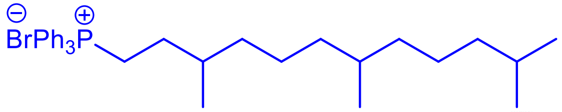

9

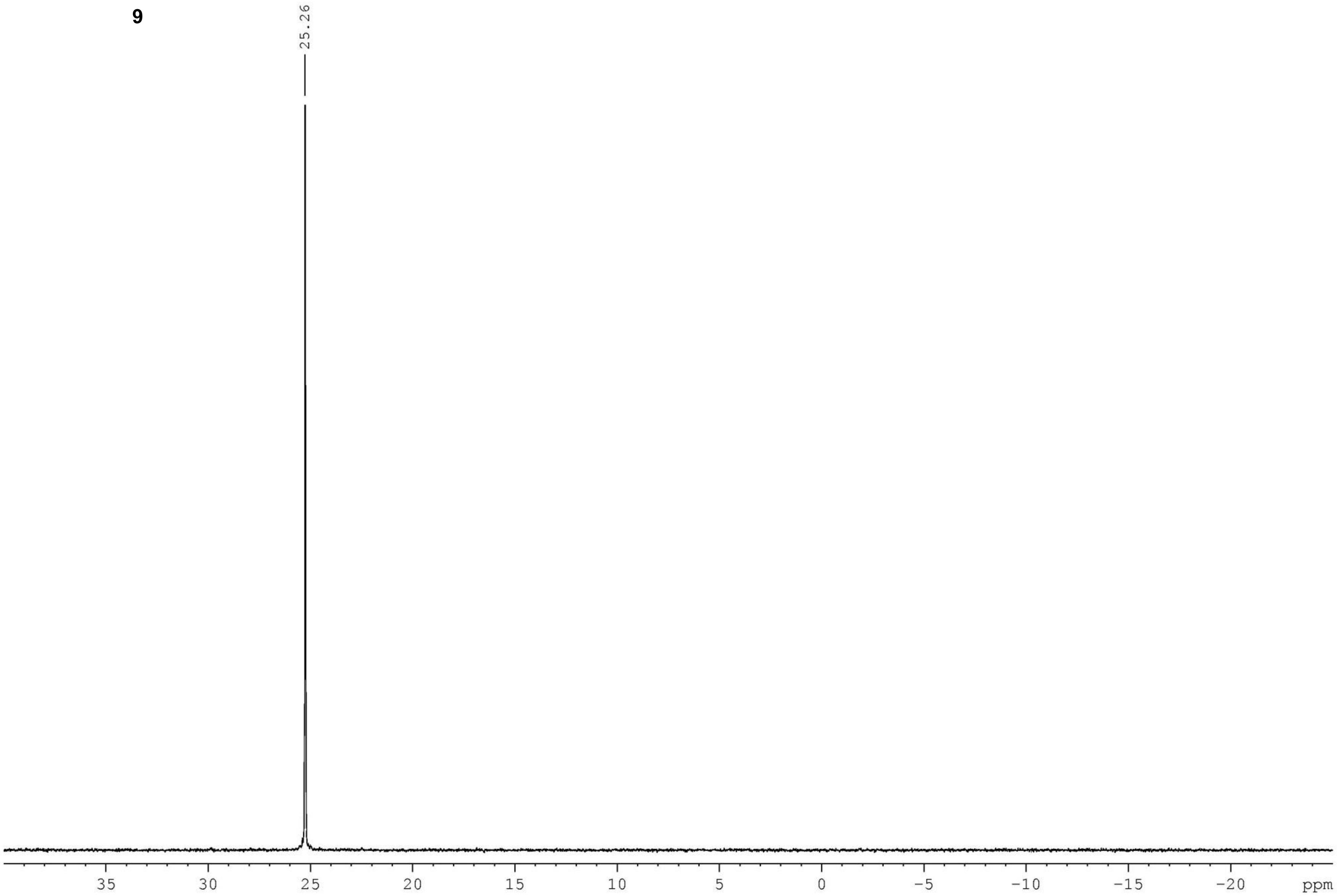

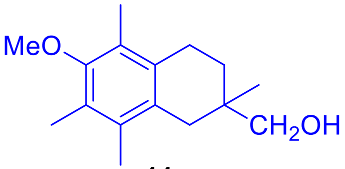
MeO
CH2OH

11

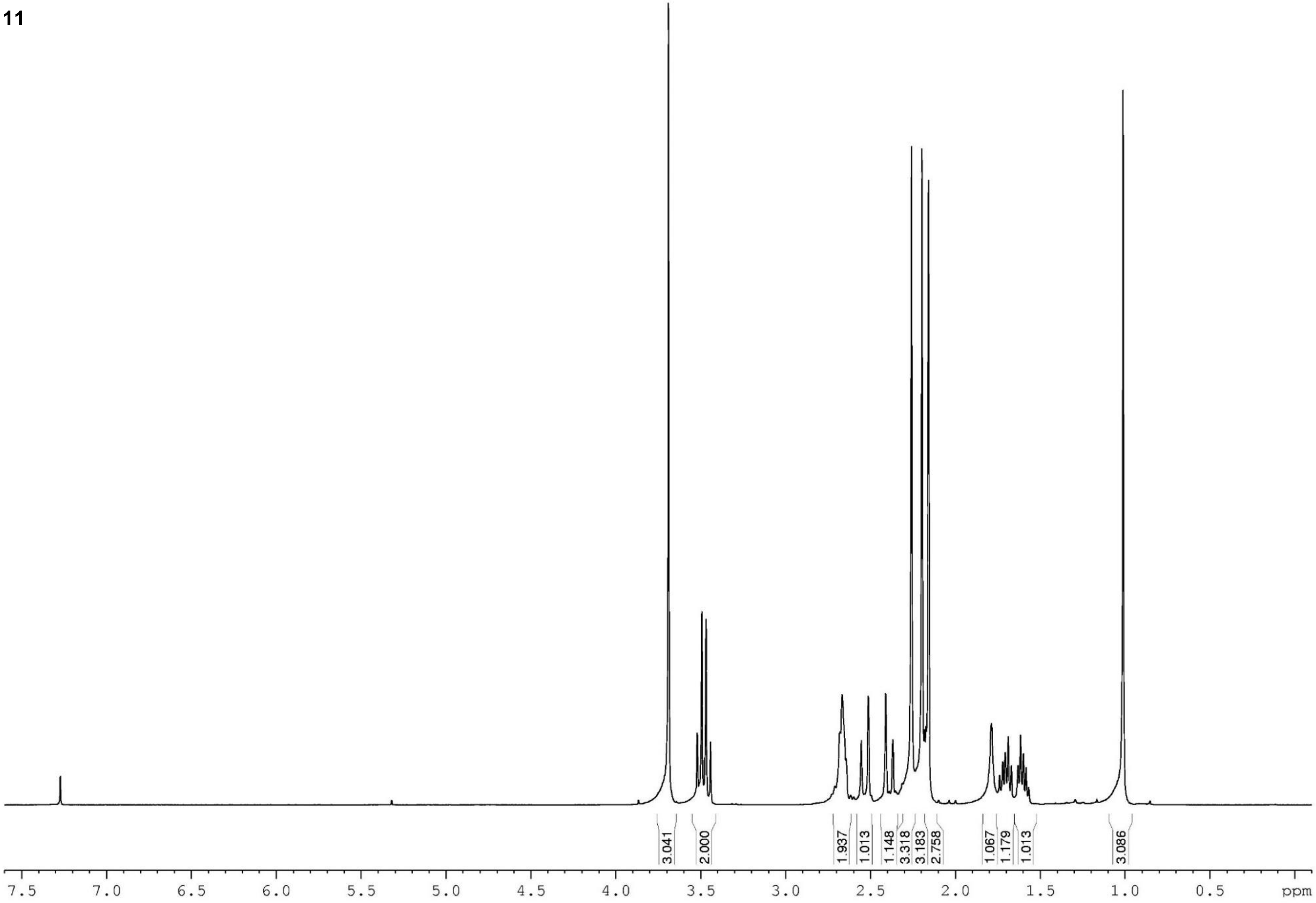
3.041
2.000
1.937
1.013
1.148
3.318
3.183
2.758
1.067
1.179
1.013
3.086
7.5
7.0
6.5
6.0
5.5
5.0
4.5
4.0
3.5
3.0
2.5
2.0
1.5
1.0
0.5
ppm

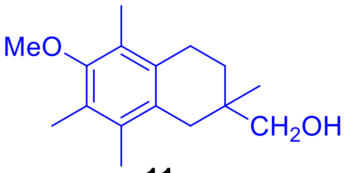
MeO
CH2OH

11

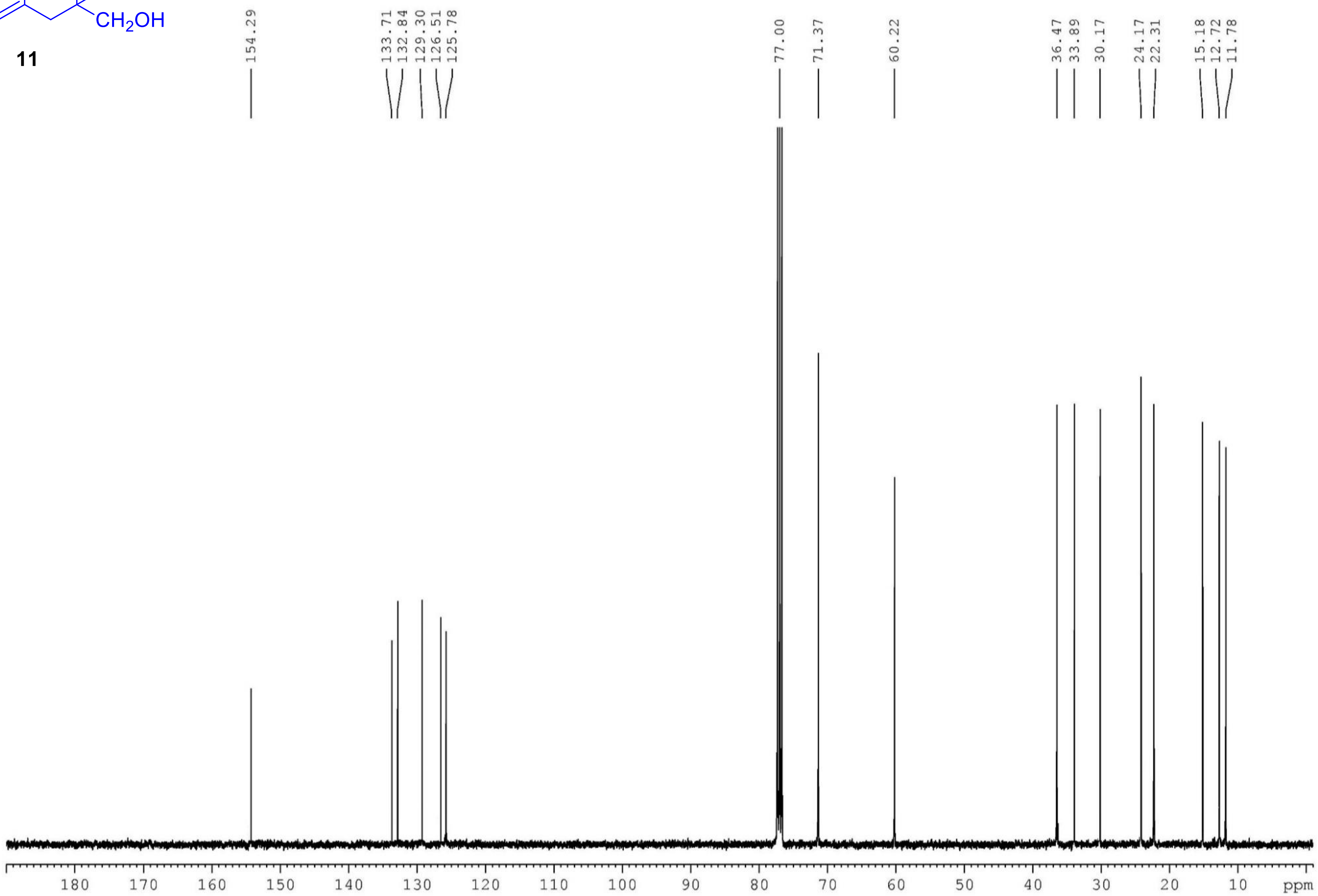
154.29
133.71
132.84
129.30
126.51
125.78
77.00
71.37
60.22
36.47
33.89
30.17
24.17
22.31
15.18
12.72
11.78
180 170 160 150 140 130 120 110 100 90 80 70 60 50 40 30 20 10 ppm

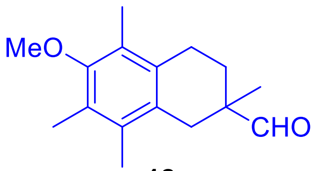
MeO
CHO

12

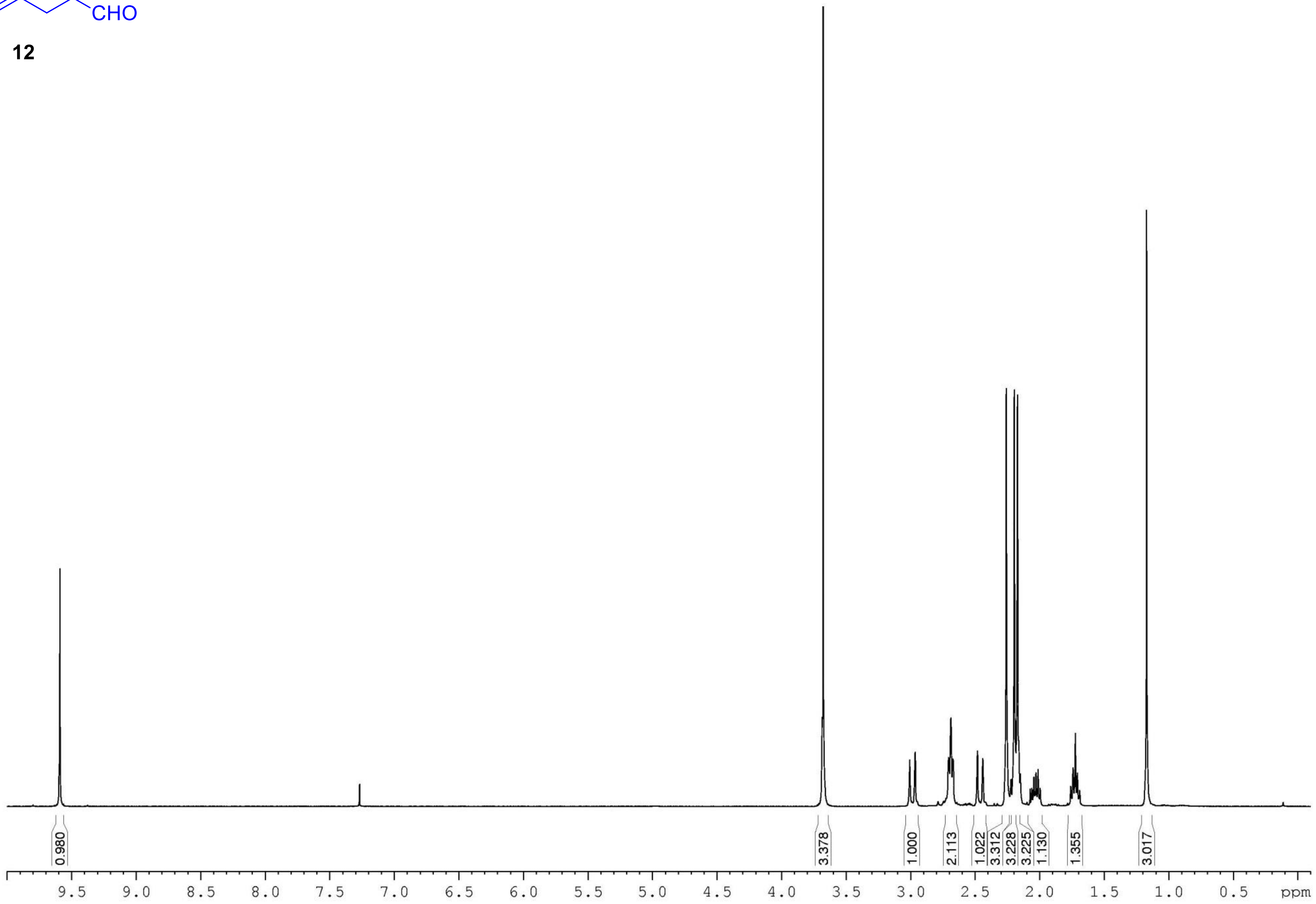
0.980
3.378
1.000
2.113
1.022
3.312
3.228
3.225
1.130
1.355
3.017
9.5 9.0 8.5 8.0 7.5 7.0 6.5 6.0 5.5 5.0 4.5 4.0 3.5 3.0 2.5 2.0 1.5 1.0 0.5 ppm

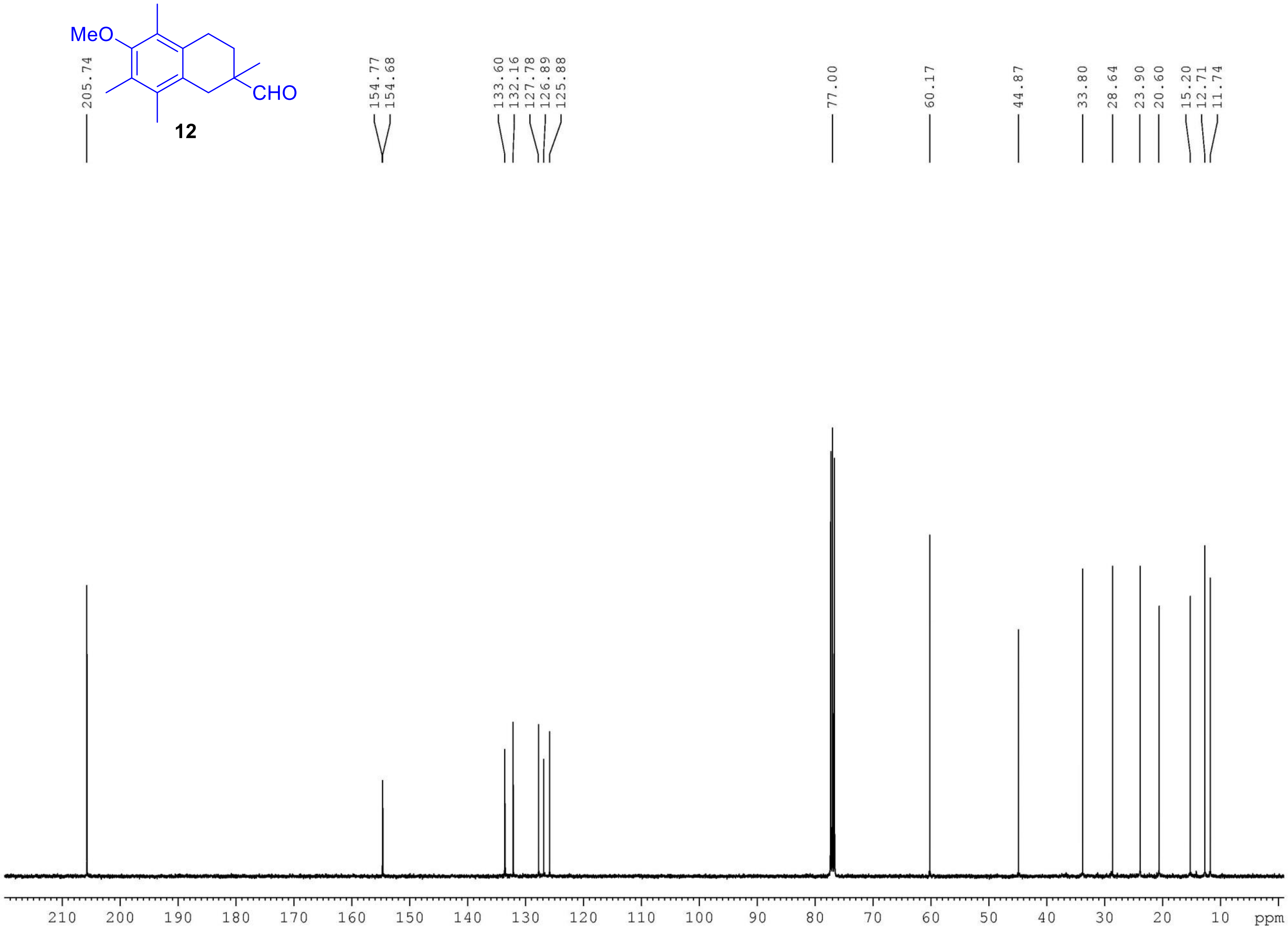
MeO
CHO
12
205.74
154.77
154.68
133.60
132.16
127.78
126.89
125.88
77.00
60.17
44.87
33.80
28.64
23.90
20.60
15.20
12.71
11.74
210 200 190 180 170 160 150 140 130 120 110 100 90 80 70 60 50 40 30 20 10 ppm

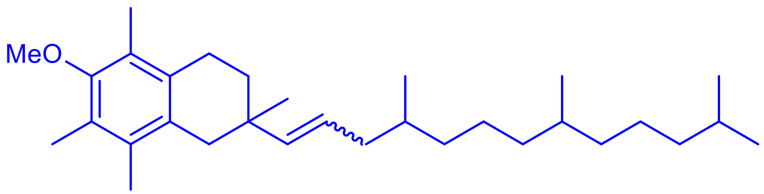
MeO

13

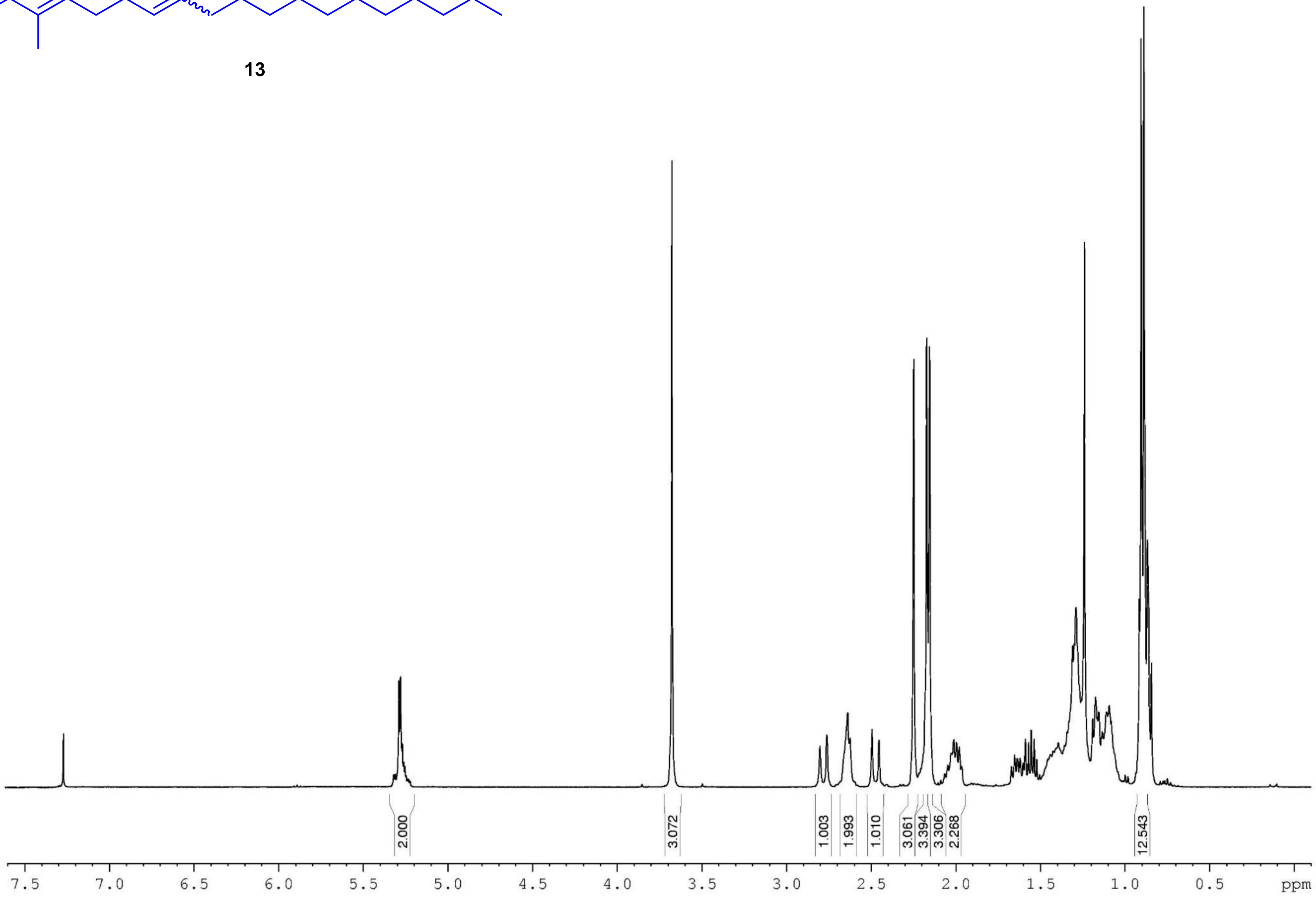
2.000
3.072
1.003
1.993
1.010
3.061
3.394
3.306
2.268
12.543
7.5
7.0
6.5
6.0
5.5
5.0
4.5
4.0
3.5
3.0
2.5
2.0
1.5
1.0
0.5
ppm

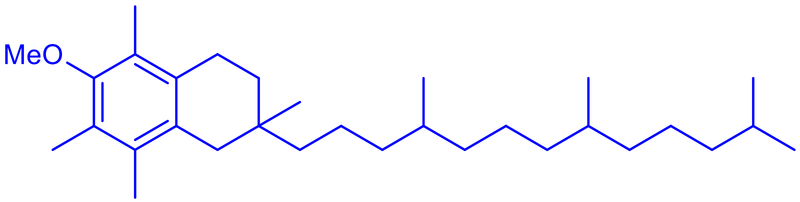
MeO

14

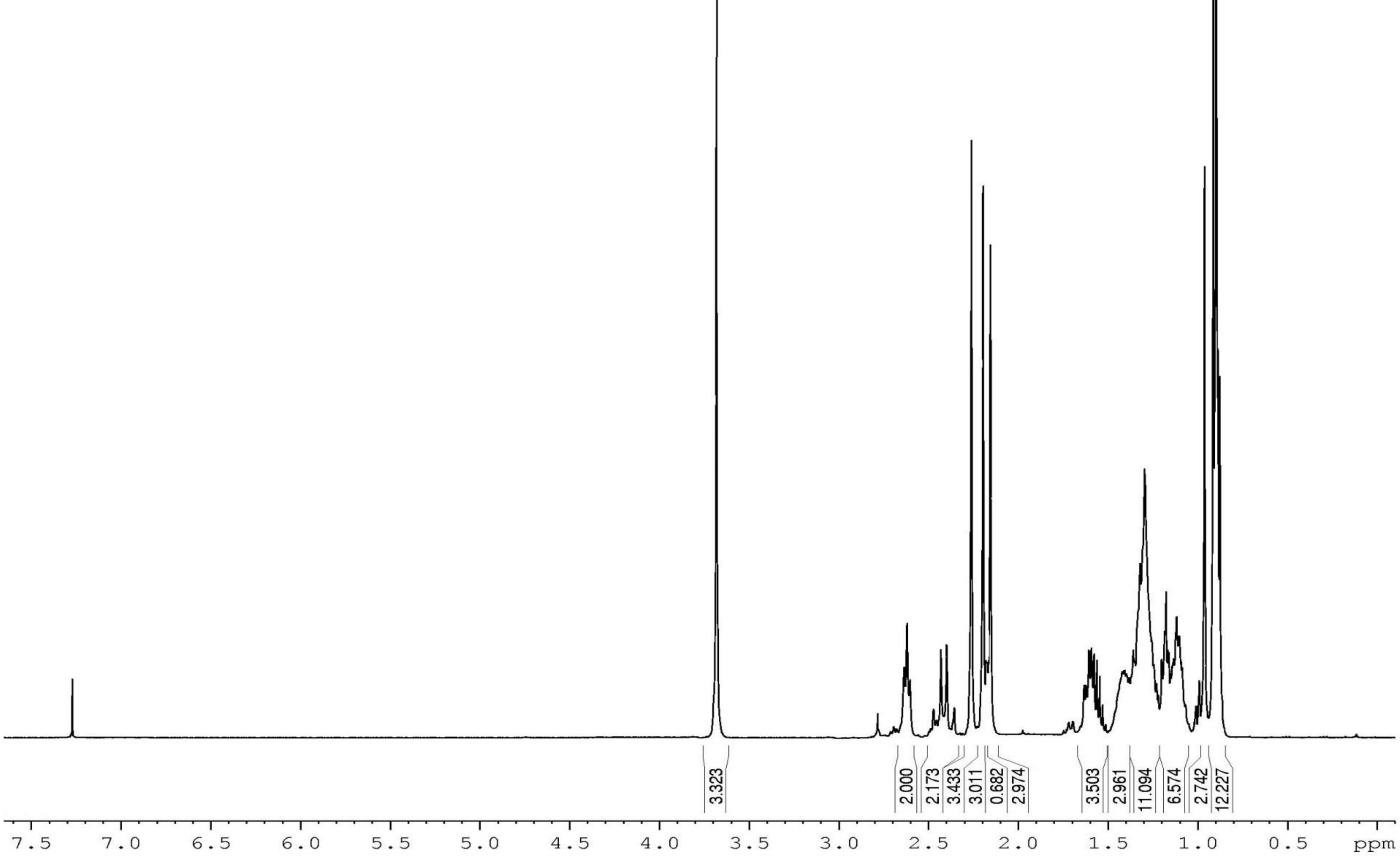
3.323
2.000
2.173
3.433
3.011
0.682
2.974
3.503
2.961
11.094
6.574
2.742
12.227
7.5
7.0
6.5
6.0
5.5
5.0
4.5
4.0
3.5
3.0
2.5
2.0
1.5
1.0
0.5
ppm
